# Hijacking the unfolded protein response (UPR) pathway: Balancing viral infection and host cell survival

**DOI:** 10.1016/j.jbc.2025.111040

**Published:** 2025-12-11

**Authors:** Haoyu Chen, Duxuan Liu, Jing Hua, Mingjie Wu, Yanhong Hua, Chenwei Feng, Zhen He, Peter Moffett, Kun Zhang

**Affiliations:** 1 College of Plant Protection, Yangzhou University, Yangzhou, Jiangsu Province, P.R. China; 2 Centre SÈVE, Département De Biologie, Université de Sherbrooke, Sherbrooke, Québec, Canada; 3 Joint International Research Laboratory of Agriculture and Agri-Product Safety of Ministry of Education of China, Yangzhou University, Yangzhou, Jiangsu Province, P. R. China

**Keywords:** ER stress, unfolded protein response (UPR), virus, apoptosis, programmed cell death (PCD)

## Abstract

The endoplasmic reticulum (ER) unfolded protein response (UPR) is a conserved eukaryotic pathway crucial for restoring cellular homeostasis under ER stress. However, diverse viruses infecting mammalian or plant cells strategically hijack and manipulate the UPR pathways featuring sensor proteins IRE1, PERK, ATF6, and bZIP17/28 to promote viral replication. While UPR is designed to provide cellular adaptive functions in response to stresses, viruses can instead exploit UPR components to instead enhance viral protein folding and remodel ER membranes for viral replication. Exploiting the UPR presents a critical dilemma: while mild UPR activation facilitates virus replication and survival, excessive or prolonged activation triggers host programmed cell death (PCD), prematurely terminating infection. Navigating this UPR tightrope is central to successful infection and replication of the virus. Viruses are not passive triggers of ER stress and the UPR. Viruses have evolved sophisticated "braking mechanisms" to actively modulate UPR signaling intensity. By fine-tuning the UPR, viruses harness the beneficial aspects of the UPR while crucially preventing the activation cascade from reaching the lethal threshold that initiates PCD. By carefully controlling the UPR balance, viruses ensure host cell survival for a sufficient duration to maximize viral progeny production. This review details the intricate interactions between the cellular UPR and infecting viruses, including links to cellular clearance pathways like autophagy and ER-associated degradation (ERAD), across different viral families (*flaviviruses*, *coronaviridae*, and *potyviruses)* and hosts (plants and animals). Understanding the sophisticated viral manipulation of the UPR equilibrium reveals fundamental insights into host-pathogen co-evolution and highlights novel potential targets for antiviral strategies aimed at disrupting this delicate balance.

The endoplasmic reticulum (ER) is a crucial site for protein synthesis, folding, and modification, ensuring that proteins slated for the secretory pathway are properly folded and modified before reaching their final destinations, such as the cell membrane or being secreted outside the cell (1, 2). The ER processes are facilitated by translocons, signal peptidases, molecular chaperones, and other folding enzymes (3, 4, 5). During protein synthesis, nascent polypeptide chains slated for the ER are co-translationally transported through the secretory protein 61 (Sec61) translocation channel embedded in the ER membrane (6, 7). Luminal or secretory proteins are released into the ER lumen upon entering the ER lumen through the channel, while membrane proteins integrate into the lipid bilayer of the ER (7). When unfolded or misfolded proteins accumulate in the ER, the ER quality control (ERQC) mechanism helps maintain the quality of protein folding and trafficking in the ER and regulates cellular homeostasis (8). ERQC operates primarily through three key mechanisms: ERAD, the unfolded protein response (UPR), and autophagy (8). To ensure protein folding fidelity, the ER is equipped with a robust quality control ERQC system (5). This system is composed of several key elements, including molecular chaperones like binding immunoglobulin protein (BiP) that assist in folding, the ERAD pathway, and the UPR (9, 10) (Fig. 1). Figure 1 provides a schematic overview of the UPR core signaling network. The UPR leads to increased synthesis of ER chaperones and a temporary repression of translation. If the UPR cannot remove the misfolded proteins, they are retro-translocated to the cytosol for degradation by the proteasome *via* the ERAD pathway.

**Figure 1:**
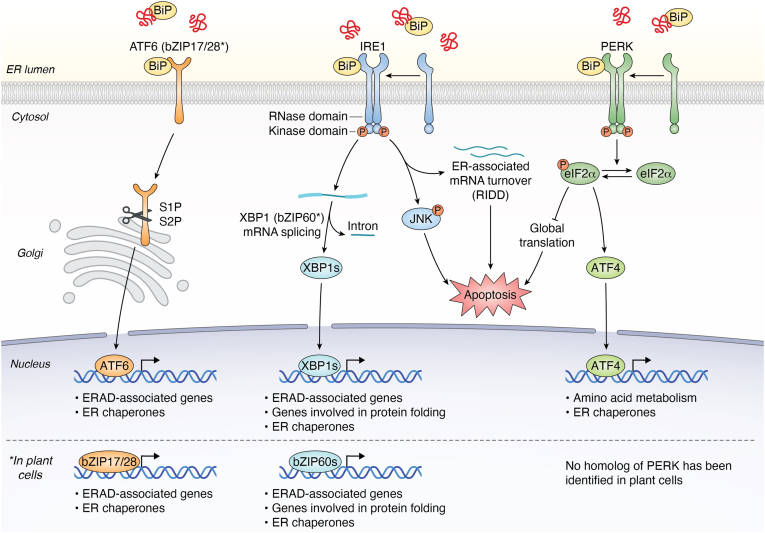
**Conserved and divergent UPR signaling pathways in animals and plants**. Upon ER stress, the chaperone BiP dissociates from the three transmembrane sensors inositol-requiring enzyme 1 (IRE1), PKR-like endoplasmic reticulum kinase (PERK), and activating transcription factor 6 (ATF6) leading to their activation. In the IRE1 pathway, dimerization and autophosphorylation, a function of its cytosolic Ser/Thr kinase domain, allosterically activates the adjacent endoribonuclease (RNase) domain. This RNase domain then performs the unconventional splicing of X-box binding protein 1 (*XBP1*) mRNA (in animals) or basic leucine zipper transcription factor 60 (*bZIP60*) mRNA (in plants) and can also initiate regulated IRE1-dependent decay (RIDD) of ER-mRNAs and activate c-Jun N-terminal kinase (JNK) signaling, potentially leading to apoptosis. Concurrently, activated PERK phosphorylates the eukaryotic initiation factor 2α (eIF2α), which inhibits global protein synthesis while selectively promoting the translation of the activating transcription factor 4 (ATF4); notably, no functional PERK homolog has been identified in plants. In the third branch, activated ATF6 translocates to the Golgi apparatus for proteolytic cleavage by S1P and S2P, releasing its cytosolic fragment which acts as a transcription factor; in plants, basic leucine zipper transcription factor 17/18 (bZIP17/28) serve as the functional homologs. Ultimately, the resulting active transcription factors (XBP1s/bZIP60s, ATF4, and the ATF6 fragment) translocate to the nucleus to upregulate a suite of genes aimed at restoring proteostasis, including those involved in protein folding and ERAD.

However, when plant or animal cells face external and internal stresses, the ERQC mechanism may become overwhelmed and unable to cope with the large accumulation of unfolded or misfolded proteins in the ER, disrupting ER homeostasis and ultimately leading to ER stress (11, 12). To complete their whole life-cycle, from entry into the host cell to final assembly and release, viruses often rely fully on various host cell organelles, particularly the ER (13). As one of the major membrane structures within the cell, the ER plays a critical role not only in the synthesis, folding, modification, and transport of lipids and proteins, but also provides a favorable microenvironment for viral replication due to its extensive surface area and dynamic membrane rearrangement capabilities (13).

This review systematically examines the intricate interplay between viruses and the host UPR. We first establish a foundational understanding of the core UPR signaling pathways in both animals and plants. Following the introduction into the UPR, we explore the mechanisms by which viruses trigger the UPR, such as imposing a high protein load and disrupting lipid homeostasis and subsequently exploit this response to facilitate viral replication through ER membrane expansion and enhanced protein folding. The review then shifts to the viral manipulation of UPR-associated networks, detailing how viruses hijack the ERAD pathway to evade immune surveillance and modulate autophagy and apoptosis to balance propagation with host cell survival. We then address the sophisticated "braking" mechanisms viruses employ to suppress the UPR and avert premature infected cell death. Finally, the review contrasts the adaptive strategies of animal and plant viruses and provides an outlook on promising future research directions in this field.

## ER function and unfolded protein response in animals and plants

Once newly synthesized proteins enter the ER, enzymes in the organelle initiate post-translational modifications, assisting in protein folding and assembly. For instance, the oligosaccharyl transferase (OST) complex attaches glycans to proteins, while oxidoreductases form, break, and rearrange disulfide bonds (14, 15). Molecular chaperones in the ER, such as BiP (also known as GRP78), a type of HSP70 ATPase, interact with folding proteins to prevent aggregation and maintain solubility (16). Fully folded proteins are then packaged into coat protein complex II (COPII)-coated vesicles and leave the ER, traveling through the classical secretory pathway to reach other cellular destinations or the plasma membrane for secretion (17). Additionally, the ER regulates calcium homeostasis through calcium channels and pumps in the membrane, which directly communicate with calcium channels on the plasma membrane (18). In the ER, calcium serves as a cofactor for chaperones like calnexin and calreticulin, playing a vital role in the folding of glycoproteins and protein quality control (19). The ER is also responsible for lipid synthesis, including fatty acids, phospholipids, sphingolipids, and components or signaling molecules (20).

The UPR is a conserved stress response that features ER resident signaling proteins that recognize disruptions in the ER and safeguards ER homeostasis by coordinating protein folding, degradation, and metabolic adaptation (21, 22, 23). While its core function is shared across kingdoms, its architecture reflects evolutionary complexity. Yeast relies solely on the ancient inositol-requiring enzyme 1 (IRE1) signaling pathway (24), whereas plants evolved a two-branch system, adding basic leucine zipper transcription factor 17/18 (bZIP17/28) as activating transcription factor 6 (ATF6)-like transcription factors (11). Mammals further expanded the UPR with three signaling pathways detailed below and illustrated in Figure 1: IRE1, PKR-like endoplasmic reticulum kinase (PERK), and ATF6 (25). Notably, both ATF6 in animals and bZIP17/28 in plants employ regulated intramembrane proteolysis, underscoring functional conservation despite sequence divergence.

IRE1 is the most conserved UPR sensor and is found in animals, plants, and yeast (26). As depicted in the IRE1 branch of Figure 1, upon dissociation from BiP, IRE1 dimerizes and activates its RNase domain to facilitate X-box binding protein 1 (*XBP1*) mRNA splicing in mammals or basic leucine zipper transcription factor 60 (*bZIP60*) mRNA in plants, producing potent transcription factors (XBP1s or bZIP60s) that induce the expression of genes involved in protein folding, ER expansion, and ERAD (27, 28, 29, 30, 31). In addition, IRE1 can trigger regulated RIDD, degrading ER-associated mRNAs to reduce the protein load (32) (Fig. 1). While this pathway supports adaptation, chronic ER stress may cause IRE1 hyperactivation, leading to cleavage of other RNAs, suppression of anti-apoptotic signals, and promotion of inflammation and apoptosis (33, 34, 35, 36).

The PERK pathway is activated when unfolded proteins titrate BiP away from PERK, enabling its dimerization and autophosphorylation (37, 38) (Fig. 1). Activated PERK phosphorylates eukaryotic initiation factor 2α (eIF2α), which suppresses general translation but permits selective translation of activating transcription factor 4 (ATF4), a transcription factor that induces stress response and antioxidant genes (39) (Fig. 1). PERK also contributes to oxidative defense through nuclear factor erythroid 2-related factor 2 (NRF2) activation (40). However, prolonged or excessive signaling drives a switch toward apoptosis, with ATF4 upregulating pro-apoptotic factors; thus, sustained PERK activity is recognized as a potent pro-death signal (33, 41, 42). This duality highlights PERK as a central rheostat between adaptation and apoptosis.

ATF6, like IRE1 and PERK, is normally bound by BiP, but when ER stress occurs, it undergoes proteolytic activation essential for resolving protein-folding stress. When stress occurs, BiP dissociates from ATF6, allowing it to translocate to the Golgi apparatus (Fig. 1). There, ATF6 is cleaved by the enzymes S1P and S2P to release an active transcription factor that moves into the nucleus (43, 44, 45) (Fig. 1). This transcription factor then activates UPR target genes, such as BiP and components of ERAD, which help to resolve protein-folding stress and clear misfolded proteins (43, 45). This pathway shares similarities with IRE1 and PERK in its role of alleviating ER stress, but its distinct proteolytic mechanism highlights its unique contribution to cellular recovery. In plants, this pathway is mediated by the bZIP17 and bZIP28 homologs, which similarly dissociate from BiP, move to the Golgi, and get cleaved to release active transcription factors that regulate UPR gene expression (46, 47) (Fig. 1).

## Mechanisms of UPR activation and viral exploitation strategies

Many positive-sense single-stranded RNA [ss(+)RNA] viruses remodel the ER to create membrane-bound replication organelles that protect viral RNA and support assembly. Examples include brome mosaic virus (BMV), potato virus X (PVX), tobacco etch virus (TEV), cowpea mosaic virus (CPMV), tomato bushy stunt virus (TBSV) (48, 49, 50, 51, 52), and flaviviruses such as dengue virus (DENV) and Zika virus (ZIKV) (53, 54, 55, 56). While essential for replication, these ER modifications impose a biosynthetic burden that frequently results in ER stress (57). To restore proteostasis, eukaryotic cells activate the UPR, a conserved signaling program that increases chaperone expression (58, 59), promotes ER-associated degradation (60), attenuates translation, and enhances ER biogenesis (11, 61, 62, 63). Under mild stress, the UPR alleviates ER overload (62), but under prolonged or severe stress, it can trigger autophagy and programmed cell death (PCD) (61, 64, 65). Thus, during viral infection, the UPR can act either as a host defense or as a vulnerability for viral exploitation.

**Figure 2:**
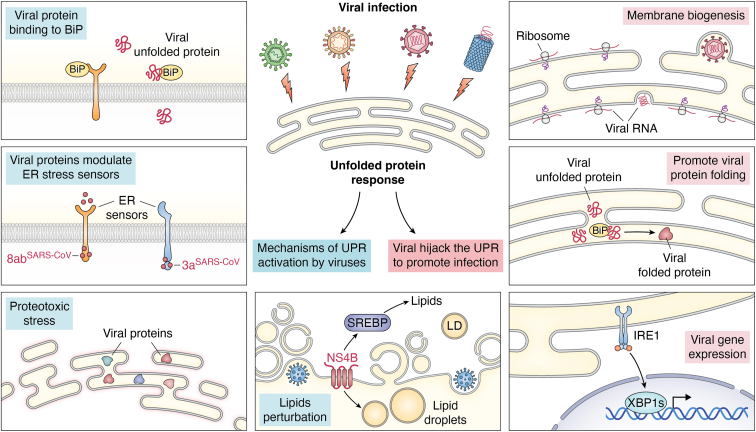
**Viral strategies for activating and exploiting the UPR**. Viruses employ a two-pronged strategy to first robustly activate the UPR and then systematically exploit its pathways to support their replication cycle. The activation mechanisms (*left panel*) are initiated by both general metabolic stress and targeted molecular interactions. General stress arises from the overwhelming demand of viral protein synthesis, which creates proteotoxic stress, and from the perturbation of host lipid metabolism linked to viral replication. In parallel, viruses utilize specific proteins for direct and precise manipulation, such as sequestering the master chaperone BiP with unfolded viral glycoproteins (HCV E1/E2) or directly binding to and modulating the core UPR sensors, as exemplified by severe acute respiratory syndrome coronavirus (SARS-CoV) proteins. Once the UPR is active (*right panel*), viruses repurpose its signaling outputs to establish a pro-viral state. Key exploitation strategies include hijacking the IRE1-XBP1s axis to drive membrane biogenesis, thereby expanding the ER and constructing membranous scaffolds essential for viral replication factories. Additionally, viruses co-opt the upregulated host chaperone machinery to ensure the correct folding and assembly of their own proteins, and can even commandeer UPR transcription factors like XBP1s to directly bind to viral promoters and enhance the expression of viral genes.

This dual nature of the UPR raises a central question: how do viruses activate this pathway and subsequently redirect it for their own benefit? As illustrated in Figure 2, viral infections can initiate UPR signaling either indirectly, when the massive synthesis of viral proteins overwhelms the folding capacity of the ER, or directly, when specific viral proteins interact with UPR sensors. Once initiated, the three canonical branches of the UPR provide multiple points of viral intervention: In some cases, the response is steered toward adaptive outputs that sustain protein synthesis and replication, whereas in others, prolonged stress responses are exploited to induce autophagy or cell death, thereby supporting dissemination or persistence (Fig. 2).

## Mechanisms by which viruses activate the UPR

To establish a successful infection, viruses have evolved multiple strategies to actively remodel the host cellular environment, with UPR induction being a critical step. These mechanisms can be broadly categorized into two main types: (i) indirect induction by overwhelming host homeostatic systems and (ii) direct activation through specific interactions between viral proteins and host factors (Fig. 2).

First, viruses indirectly trigger the UPR by placing an immense burden on the ER's metabolic capacity. In both plant and animal cells, the massive synthesis of viral proteins inherently challenges the protein-folding machinery of the ER leading to the accumulation of unfolded or misfolded proteins (Fig. 2). For example, in the coronaviridae family, the spike protein is extensively folded and glycosylated within the ER, which places a significant folding burden on the ER and contributes to ER stress (66, 67, 68). Simultaneously, viral infection disrupts lipid homeostasis, a known trigger for the UPR (Fig. 2). Lipid disruption occurs through extensive intracellular membrane remodeling to create viral replication sites and through specific viral actions, such as in hepatitis C virus (HCV) infection, where the core and NS5A proteins, and NS4B translocate to lipid droplets, disrupting the ER-lipid droplet interface (69, 70) (Fig. 2). This indirect activation of the UPR *via* systemic stress is a conserved strategy, as evidenced by the general upregulation of the chaperone BiP in plants infected with viruses like turnip mosaic virus (TuMV) and oilseed rape mosaic virus (ORMV) (71, 72). The combination of proteotoxic and lipotoxic stress makes UPR activation an inevitable consequence of the high demands of viral replication.

Beyond the general stresses, viruses employ more direct and specific mechanisms to ensure UPR activation. One such strategy involves viral proteins directly engaging the core UPR sensors. For example, the severe acute respiratory syndrome coronavirus (SARS-CoV) accessory protein 8 ab binds directly to the luminal domain of ATF6, while its 3a protein selectively activates the PERK pathway (73) (Fig. 2). Another common tactic is the sequestration of the master UPR regulator, BiP. The accumulation of unfolded viral glycoproteins, such as the G protein of vesicular stomatitis virus (VSV), the hemagglutinin-neuraminidase (HN) of paramyxovirus (PMV) SV5, the hemagglutinin of Influenza A virus (IAV), and the E1/E2 proteins of HCV, can titrate BiP away from the sensors, leading to their activation (74, 75) (Fig. 2). Furthermore, viruses can interfere with the broader chaperone network to induce the UPR. A clear example is the HCV NS5A protein, which recruits co-chaperones FK506-binding protein 8 (FKBP8) and human (h) butyrate-induced transcript 1 (hB-ind1) into the replication complex, disrupting the function of the heat shock protein 90 (HSP90) chaperone system and thereby impairing protein folding, which in turn triggers the UPR (76, 77, 78, 79) (Fig. 2). This principle of using specific viral proteins as targeted UPR elicitors is also well-documented in plants. For instance, the potato virus X (PVX) movement protein triple gene block protein 3 (TGBp3) and the second 6 kDa protein (6K2) of TuMV are known inducers of the plant UPR (80, 81, 82). While a direct protein-sensor binding may not occur, the mechanism is highly specific; 6K2, for example, selectively activates the IRE1-bZIP60 pathway as a direct consequence of its function in remodeling the ER to build replication factories (82, 83).

Collectively, through both broad and targeted approaches, viruses robustly activate the UPR, transforming it from a host defense system into a pro-viral cellular state. Having established how viruses "turn on" the UPR, the following section will explore how they exploit these activated pathways to facilitate their lifecycle.

## Viral exploitation of the UPR to remodel the host environment for infection

The activation of the UPR represents a critical turning point in infection, where viruses pivot from inducing stress to actively exploiting the resulting cellular response. Viruses systematically repurpose the UPR from a host-protective mechanism into a pro-viral program that remodels the cellular environment. This exploitation manifests in several well-documented strategies, most notably: (i) reconfiguring host membranes into scaffolds for viral replication, (ii) co-opting the protein folding and chaperone machinery to manage the high viral protein load, and (iii) hijacking UPR signaling components to directly promote viral gene expression (Fig. 2).

### Alterations in membrane biogenesis

A primary advantage conferred by the UPR is the extensive remodeling of host cell membranes into platforms essential for viral replication and assembly. XBP1 is a transcription factor that upregulates UPR-related genes and promotes the biosynthesis of the ER and Golgi network. Flaviviruses, such as Japanese encephalitis virus (JeV), DENV, and HCV, have enveloped viral particles and encode glycoproteins that accumulate along the ER-Golgi network (84). After replication, the genome is encapsidated, and viral particles mature by budding through these cellular membranes (79). In the early stages of viral infection, flaviviruses trigger the IRE1/XBP1 arm of the UPR to reduce the cytotoxicity of viral infection (84) (Fig. 2). DENV, HCV, and West Nile virus (WNV) stimulate fatty acid synthesis, which is essential for viral replication and maturation (79). Therefore, viruses require the XBP1 pathway of the UPR to regulate ER expansion, reducing ER stress caused during viral maturation.

### Modulation of protein folding

Viruses extensively co-opt the UPR to augment the host's protein folding capacity, thereby ensuring the fidelity and assembly of their own structural and non-structural proteins. Many viruses, particularly within the *faviviridae* family, induce canonical UPR to increase the expression of key ER chaperones. For instance, the HCV envelope glycoproteins (E1 and E2) bind to chaperones like BiP, calreticulin, and calnexin; the E2 protein alone is sufficient to trigger all three UPR branches and elevate BiP transcription (79). Similarly, the spike protein of SARS-CoV enhances BiP/Grp78 levels *via* the PERK pathway (85). This viral control can also be highly specific. African swine fever virus (ASFV), for example, selectively activates only the ATF6 branch to precisely upregulate ER chaperones, including calreticulin and calnexin (86) (Fig. 2). This fundamental strategy is conserved in plant viruses, where the 6K2 protein of TuMV and the TGB3 protein of PVX both activate the analogous bZIP60 pathway to increase BiP expression (22) (Fig. 2).

### Regulation of gene expression

Beyond supporting macromolecular synthesis, viruses can hijack core components of the UPR machinery to serve as direct transcriptional regulators for their own genomes. In cells infected with adenovirus (ADV), the splicing of *XBP1u* to *XBP1s* mRNA is enhanced, and the viral glycoprotein E3-19K promotes the expression of early viral genes by binding *XBP1s* to the E1 and E4 promoters, as confirmed by chromatin immunoprecipitation and E1 promoter mutation (87) (Fig. 2). A similar phenomenon was observed in studies of cytomegalovirus, where the early gene expression of mouse cytomegalovirus (MCMV) was inhibited by X*BP1u* (88). MCMV briefly activates the IRE1-XBP1 branch, depletes *XBP1u*, and relieves the transcriptional repression on the early viral promoters to promote viral replication (88) (Fig. 2).

### Viruses evoke different strategies to activate the UPR

Overall, viruses have evolved sophisticated strategies to co-opt and manipulate the UPR machinery, creating a cellular environment conducive to advancing their life cycle. The following are examples from animal and plant viruses that illustrate the diverse mechanisms employed to engage with this fundamental host stress response. The following examples from animal and plant viruses, detailed in Tables 1 and 2, illustrate how these principles are applied.

**Table 1:** UPR pathways induced by animal viruses

Family/genus	Virus	UPR pathway	Functions	Reference
*Flaviviridae*/ *Hepacivirus*	Hepatitis C virus (HCV)	IRE1/PERK/ATF6	NS4B interacts with ATF6 to activate UPR, E1 binds to PERK to repress UPR.	(79, 110)
*Flaviviridae*/ *Flavivirus*	West Nile virus (WNV)	IRE1/PERK/ATF6	NS4B activates UPR, particularly IRE1 pathway	(157)
*Flaviviridae*/ *Flavivirus*	Japanese encephalitis virus (JeV)	IRE1/PERK/ATF6	trigger the UPR-induced pro-apoptotic transcription factor CHOP (CCAAT/enhancer-binding protein homologous protein)	(123)
*Flaviviridae*/ *Flavivirus*	Dengue virus (DENV)	IRE1/PERK/ATF6	NS4B activates UPR, particularly IRE1 pathway	(137, 139, 158)
*Flaviviridae*/ *Flavivirus*	Tick-borne encephalitis virus (TBEV)	IRE1/ATF6	TBEV manipulate the UPR to facilitate its own replication	(159)
*Flaviviridae*/ *Flavivirus*	Zika virus (ZIKV)	IRE1/PERK/ATF6	Protein E of ZIKV interacts with GRP78	(160, 161, 162)
*Flaviviridae*/ *Pestivirus*	Classical swine fever virus (CSFV)	IRE1/PERK/ATF6	Up-regulates the unfolded protein response to promote its replication especially the IRE pathway	(163)
*Coronavidae/* *Betacoronavirus*	Severe acute respiratory syndrome coronavirus (SARS-CoV)	PERK	Spike protein induces UPR through PERK, ORF3a induces ER stress	(85)
*Coronavidae/* *Betacoronavirus*	Severe acute respiratory syndrome coronavirus-2 (SARS-CoV-2)	IRE1/PERK/ATF6	Spike protein: Folding in ER may induce ER stress and activate UPR, supporting viral replication.	(66, 67, 68)
*Coronavidae/* *Betacoronavirus*	Murine hepatitis virus (MHV)	IRE1/PERK/ATF6	Spike protein induces UPR	(66, 164)
*Coronavidae/* *Gammacoronavirus*	Infectious bronchitis virus (IBV)	IRE1/PERK/ATF6	Spike protein induces UPR	(165)
*Coronavidae/* *Alphacoronavirus*	Transmissible gastroenteritis virus (TGEV)	IRE1/PERK/ATF6	Spike protein induces UPR	(166)
*Coronavidae/* *Alphacoronavirus*	Porcine epidemic diarrhea virus (PEDV)	IRE1/PERK/ATF6	Spike protein induces UPR	(167, 168)
*Coronavidae/* *Alphacoronavirus*	Human coronaviruses (HCoV)	IRE1	-	(169)
*Coronavidae/* *Deltacoronavirus*	Porcine deltacoronavirus (PDCoV)	IRE1/PERK/ATF6	-	(170)
*Coronavidae/* *Betacoronavirus*	Middle East respiratory syndrome CoV (MERS-CoV)	PERK	-	(171)
*Arteriviridae*/ *Porartevirus*	Porcine reproductive and respiratory syndrome virus (PRRSV)	IRE1/PERK	The glycoprotein GP2a contributes to the GRP78 decay *via* proteasomal pathway	(172)
*Filoviridae*/ *Ebolavirus*	Ebola virus (EBOV)	IRE1/PERK	-	(173)
*Filoviridae*/ *Marburgvirus*	Marburg virus (MARV)	IRE1/PERK	-	(174)
*Orthomyxoviridae*/ *Alphainfluenzavirus*	Influenza A virus (IAV)	IRE1/PERK/ATF6	Hemagglutinin (HA): HA folding in ER causes ER stress, activating IRE1-XBP1s to enhance ER capacity for viral replication.	(175)
*Adenoviridae*/ *Mastadenovirus*	Adenovirus (AdV)	IRE1	The E3-19K lumenal domain activates the IRE1α nuclease, which initiates mRNA splicing of X-box binding protein-1 (XBP1).	(87)
*Poxviridae*/ *Orthopoxvirus*	Vaccinia virus (VACV)	ATF6	-	(176)
*Herpesviridae/Betaherpesvirus*	Human cytomegalovirus (HCMV)	IRE1/PERK/ATF6	UL148 and US11 induces UPR	(177)
*Herpesviridae/Gammaherpesvirus*	Kaposi’s sarcoma herpes virus (KSHV)	IRE1/PERK/ATF6	vGPCR activates UPR	(178, 179)
*Herpesviridae/Alphaherpesvirus*	Herpes simplex virus type 1 (HSV-1)	PERK/ATF6	-	(179, 180)
*Herpesviridae/Alphaherpesvirus*	Varicella zoster virus (VZV)	IRE1	-	(181)
*Herpesviridae*/Herpesviridae	Pseudorabies virus (PRV)	IRE1/PERK	-	(182)
*Hepadnaviridae*/ *Orthohepadnavirus*	Human hepatitis B virus (HBV)	IRE1	-	(183)
*Picornaviridae*/ *Senecavirus*	Seneca valley virus (SVV)	PERK/ATF6	-	(184)
*Paramyxoviridae/Senecavirus*	Newcastle disease virus (NDV)	PERK/ATF6	-	(185)
*Retroviridae/Lentivirus*	Human immunodeficiency virus 1 (HIV)	IRE1/PERK/ATF6	Vpr activates PERK pathway	(186)
*Herpesviridae/Gammaherpesvirinae*	Epstein-Barr virus (EBV)	IRE1	*BRLF1* activated by XBP1s	(187)
*Asfarviridae/Asfivirus*	African swine fever virus (ASFV)	ATF6	-	(86)

**Table 2:** UPR pathways induced by plant viruses

Family/genus	Virus	UPR pathway	UPR related protein	Reference
*Phenuiviridae/* *Tenuivirus*	Rice stripe virus (RSV)	bZIP17/28	NSvc2/NSvc4 induces UPR	(153)
*Alphaflexiviridae/Potexvirus*	Potato virus X (PVX)	bZIP60	TGBP3 induces UPR	(188)
*Potyviridae/* *Potyvirus*	Sugarcane streak mosaic virus (SCSMV)	bZIP60	6K2 induces UPR	(152)
*Potyviridae/* *Potyvirus*	Potato virus Y (PVY)	bZIP60	6K2 induces UPR	(188)
*Alphaflexiviridae/Potexvirus*	Plantago asiatica mosaic virus (PlAMV)	bZIP60	TGBP3 induces UPR	(189)
*Potyviridae/potyvirus*	Turnip mosaic virus (TuMV)	bZIP60	6K2 induces UPR	(189)
*Benyviridae* */Benyvirus*	Beet necrotic yellow vein virus (BNYVV)	bZIP17/28	-	(190)
*Alphaflexiviridae/Allexivirus*	Garlic virus X (GarVX)	bZIP60	P11 induces UPR	(191)
*Reoviridae*/*Fijivirus*	Rice black-streaked dwarf virus (RBSDV)	bZIP60	P10 induces UPR	(192)
*Virgaviridae/* *Tobamovirus*	Tobacco mosaic virus (TMV)	bZIP60; bZIP17/28	-	(193)
*Bromoviridae/* *Cucumovirus*	Cucumber mosaic virus (CMV)	bZIP60; bZIP17/28	-	(193)
*Closteroviridae/Crinivirus*	Lettuce infectious yellows virus (LIYV)	bZIP60	-	(194)
*Closteroviridae/Closterovirus*	Citrus tristeza virus (CTV)	bZIP60	-	(194)
*Geminiviridae/Begomovirus*	Tomato yellow leaf curl virus (TYLCCNV)	bZIP60; bZIP17/28	βC1 activates bZIP60; βV1 activates bZIP17/28	(155, 156)

Specifically, viruses in animal hosts (Table 1) demonstrate these activation mechanisms and exploitation strategies. *Flaviviridae* (HCV, DENV, ZIKV, CSFV) often activate all UPR branches *via* indirect stress (viral glycoprotein accumulation) and direct engagement (NS4B or NS5A) (Table 1). They primarily rely on the IRE1–XBP1 axis for membrane biogenesis remodeling and protein folding modulation, while PERK and ATF6 synergistically attenuate translation and hijack gene expression, supporting viral replication, immune evasion, and chronic infection (Table 1). *Coronaviridae* (SARS-CoV, SARS-CoV-2, MERS-CoV) mainly hijack the PERK–eIF2α pathway through indirect folding burdens (spike) and direct activation (8ab/3a) to inhibit host translation, optimizing viral protein synthesis and protein folding, thereby promoting viral assembly and dissemination (Table 1). *Herpesviridae* (HCMV, KSHV, HSV-1), AdV, and ASFV prioritize activating ATF6 or IRE1 *via* direct binding of viral proteins (UL148/US11, vGPCR, EP152 R/MGF110-7L) for precise protein folding modulation and gene expression hijacking, with membrane remodeling *via* autophagy/apoptosis also facilitating persistent infection (Table 1).

Similarly, viruses in plant hosts (Table 2) exhibit the application of these mechanisms and strategies. Viruses from genera like *Potyvirus* (TuMV, PVY, SCSMV) and *Alphaflexivirus* (PVX, PlAMV) utilize direct activation by membrane-associated proteins (6K2 and TGBp3 binding IRE1) and the indirect stress they induce to selectively drive the IRE1–bZIP60 pathway (Table 2). This leads to membrane remodeling for expanded ER capacity, protein folding modulation for increased BiP expression supporting viral replication, and aids gene expression for stress alleviation. TYLCCNV from *Geminiviridae*, *via* direct action of βC1/βV1, activates bZIP60 or bZIP17/28, promoting protein folding and gene expression hijacking, thereby enhancing viral dissemination and immune suppression (Table 2). This selective UPR branch utilization enables viruses to precisely tune UPR outputs to match their lifecycle needs, minimizing host defense activation, and ultimately enhancing viral adaptability and pathogenicity.

## Viral manipulation of UPR-associated pathways to aid replication

The strategic activation of UPR is only the first step in a virus's manipulation of the host cell to avoid the deleterious effects of the UPR and to aid viral replication. The subsequent, and perhaps more critical challenge is to selectively control the downstream consequences of this activation. The UPR acts as a crucial decision-making hub that governs protein degradation, organelle turnover, and ultimately, cell survival or death. To maximize its replicative window, a virus must therefore navigate this complex signaling network. This section details how viruses fine-tune the UPR's outputs by: (i) hijacking the ERAD pathway to selectively eliminate antiviral host proteins while protecting their own, (ii) modulating autophagy in a delicate balance to support viral maturation without compromising the host cell, and (iii) suppressing apoptosis to avert premature cell death.

### Hijack ERAD pathway

ERAD is a conserved protein clearance mechanism present in all eukaryotic cells and serves as a crucial branch of the UPR. ERAD is a complex protein quality control system responsible for identifying and eliminating misfolded or improperly assembled proteins in the ER. The process involves four key steps: First, molecular chaperones and glycosylation modifications recognize the misfolded proteins. Next, these proteins are transferred to the cytosol through transmembrane complexes, a process known as dislocation or retro-translocation. Third, E3 ubiquitin ligases attach multiple ubiquitin molecules to the misfolded proteins, marking them for 26S proteasome-mediated degradation. Finally, the ubiquitinated proteins are recognized by the 26S proteasome and degraded into small peptides, completing the clearance process (9, 89, 90). Through this orderly mechanism, ERAD prevents the accumulation of misfolded proteins, thus maintaining cellular homeostasis and avoiding potential cytotoxicity. However, viruses have evolved sophisticated strategies to hijack this host-protective machinery for three distinct purposes: to dismantle immune surveillance, to regulate their own protein expression, and to facilitate physical entry into the cytoplasm.

**Figure 3:**
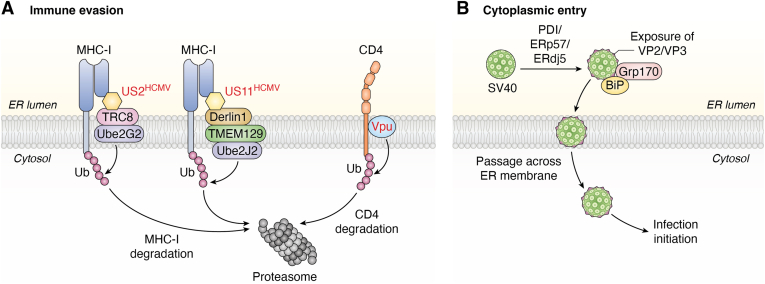
**Viruses have evolved distinct strategies to hijack the host ERAD pathway for immune evasion and physical translocation**. *A*, Immune evasion: Viral effectors co-opt ERAD machinery to target host immune surveillance proteins for premature degradation. This is exemplified by HCMV, which uses its US11 protein to remove MHC-I molecules, and by HIV-1, whose Vpu protein targets the CD4 receptor for destruction. *B*, cytoplasmic entry: Non-enveloped viruses, such as simian virus (SV40), subvert ERAD components for a different purpose. They exploit the retro-translocation machinery not for degradation but to facilitate their own physical passage across the ER membrane and into the cytosol, a critical step for initiating infection.

A primary viral strategy is to co-opt ERAD to eliminate host proteins involved in immune recognition. Several viruses use this pathway to effectively render infected cells invisible to the immune system. For instance, human cytomegalovirus (HCMV) provides a classic example of this strategy, employing two distinct viral effectors, US2 and US11, to target MHC-I molecules for degradation. Although both achieve the same outcome, they hijack different host machinery: US2 co-opts the E3 ubiquitin ligase TRC8, while US11 utilizes TMEM129 to reroute MHC-I for proteasomal destruction (91, 92) (Fig. 3*A*). Murine gammaherpesvirus 68 (MHV68) achieves a similar outcome using its virally encoded E3 ubiquitin ligase, mK3, to mark newly synthesized MHC-I for destruction (Fig. 3*A*). The human immunodeficiency virus (HIV-1) also weaponizes this pathway; its Vpu protein targets the host T-cell receptor CD4, which the virus uses for entry, for ERAD-mediated degradation (Fig. 3*A*). By removing CD4 from the cell surface, HIV-1 reduces the chance of superinfection and subsequent immune capture, thus supporting its replication (93).

Viruses also utilize ERAD to precisely control the levels of their own proteins, creating a balance that supports robust replication while avoiding cytotoxicity or immune detection. JeV and DENV, for example, use the derlin family members 2 (Derlin2)-mediated ERAD pathway to degrade their own NS4B protein, preventing its overaccumulation from disrupting the replication complex (94). Similarly, HCV and hepatitis B virus (HBV) exploit ERAD to clear excess viral glycoproteins. By upregulating ERAD components like EDEMs, these viruses not only avoid immune recognition but also promote the establishment of persistent chronic infections (95, 96).

Finally, viruses have learned to subvert the ERAD machinery for physical translocation, using its components not for degradation but to penetrate the ER membrane. The Hepatitis E virus (HEV) capsid protein undergoes retro-translocation *via* the ERAD system into the cytoplasm, yet cleverly evades subsequent proteasomal degradation, thereby facilitating viral particle assembly (97). This hijacking is even more dramatic for non-enveloped DNA viruses like polyomaviruses (PyVs). After being transported to the ER, viruses such as simian virus 40 (SV40) embed in the ER membrane and exploit key ERAD factors to facilitate their translocation into the cytoplasm, a critical step for successful infection (98, 99, 100, 101) (Fig. 3*B*). Through these diverse mechanisms, viruses have repurposed a host defense pathway into a multifaceted pro-viral tool.

### Modulate autophagy

**Figure 4:**
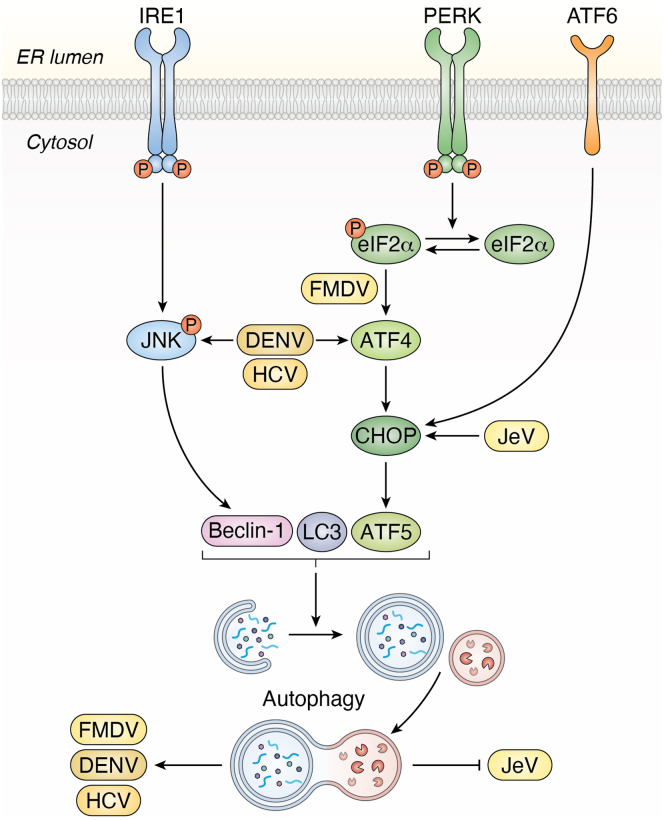
**Interplay between UPR signaling and autophagy regulation during viral infection**. Viral infection triggers ER stress, activating UPR pathways that are directly linked to the induction of autophagy. Signaling from core UPR branches, such as the PERK pathway, *via* eIF2α phosphorylation and ATF4 translation, and the IRE1 pathway, through effectors like JNK, converges to initiate autophagy. This process, however, has a dual role in viral infection. On one hand, it can act as a host defense mechanism ("Inhibit virus replication"), targeting viral components for degradation in a process known as xenophagy. On the other hand, many viruses have evolved to subvert this response ("Help virus replication"). This is exemplified by viruses like HCV, DENV, and FMDV, which are shown to induce or manipulate autophagy to create membranous scaffolds for their replication complexes and enhance overall viral production.

As a consequence of sustained ER stress, the cell often initiates autophagy, a catabolic process that degrades damaged organelles and protein aggregates to restore homeostasis and support survival (102, 103). The UPR is directly linked to autophagy induction through signaling molecules like c-Jun N-terminal kinase (JNK), XBP1, and ATF4, positioning autophagy as a critical downstream response to clear problematic proteins (Fig. 4) (104, 105, 106). This connection is conserved in plants, where the UPR sensor IRE1b is essential for inducing autophagy under ER stress (107). This pathway, however, represents a crucial battleground in the host-virus conflict. While autophagy often functions as an antiviral defense, many viruses have evolved to modulate and exploit it to facilitate their own life cycle.

In its canonical role, autophagy serves as a potent antiviral mechanism by recognizing and eliminating viral components (Fig. 4). This defense is evident in plants, where the selective autophagy receptor NBR1 binds to the P4 protein of cauliflower mosaic virus (CaMV), targeting it for degradation and thereby restricting viral infection (108). Similarly, silencing key autophagy genes (ATG5 and ATG7) in plants exacerbates infection by tomato leaf curl yunnan virus (TLCYnV), confirming the pathway's defensive function.

However, many successful viruses have learned to turn this defensive weapon against the host, repurposing autophagy to create a favorable replication environment (Fig. 4). HCV is a master manipulator of this process, using UPR regulators like PERK and IRE1α to induce autophagy and then utilizing the resulting autophagic membranes as specialized scaffolds for its RNA replication complexes (109). Moreover, HCV ensures robust autophagy by blocking the inhibitory AKT-TSC-MTORC1 pathway and even induces mitophagy to suppress apoptosis, thereby prolonging the life of its cellular factory for maximal virion production and release (109, 110, 111, 112). Other viruses employ similar strategies: DENV triggers the IRE1α-JNK branch to induce autophagy, which supports its replication and degrades immune components (113, 114) (Fig. 4), while foot and mouth disease virus (FMDV) relies on PERK-induced autophagy to enhance viral titers (115) (Fig. 4).

This viral subversion can be remarkably specific. In plants, TuMV uses its 6K2 protein to activate the IRE1/bZIP60 pathway, which upregulates the autophagy receptor NBR1. In a striking twist, instead of targeting the virus for degradation, NBR1 is co-opted to recruit the viral polymerase (NIb) and the host protein ATG8f to form the viral replication complex, using the host vacuolar membrane as a platform for viral assembly (116). In this manner, viruses do not simply induce autophagy; they skillfully modulate its machinery to build replication factories, evade immunity, and control the fate of the infected cell.

### Regulation of PCD

**Figure 5:**
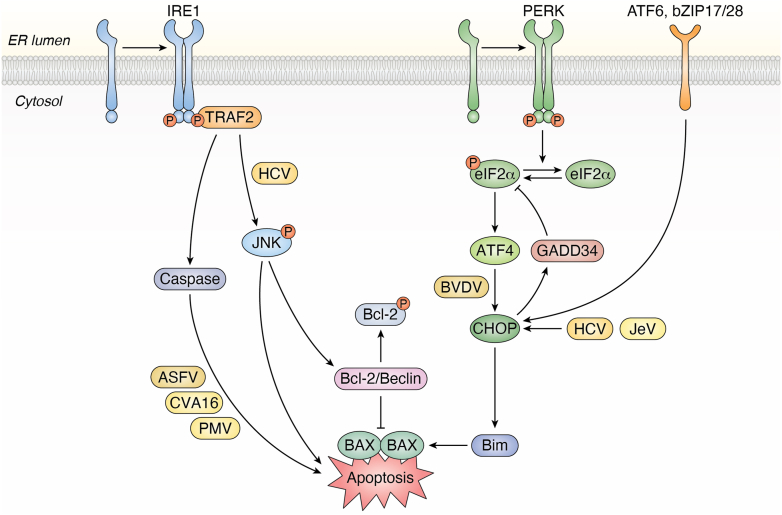
**Viral modulation of UPR-mediated apoptosis signaling**. ER stress, often induced by viral infection, activates UPR pathways that can initiate pro-apoptotic cascades. The IRE1 pathway recruits TRAF2 (an interaction modulated by HCV) to activate the JNK cascade. JNK then promotes apoptosis, culminating in caspase activation, a process influenced by viruses like ASFV, CVA16, and PMV. Concurrently, the PERK pathway leads to eIF2α phosphorylation and selective ATF4 translation. ATF4 induces the transcription factor C/EBP homologous protein (CHOP), a key mediator of apoptosis whose expression is influenced by BVDV. Signals from both the IRE1-JNK and PERK-CHOP axes converge to perturb the equilibrium of Bcl-2 family proteins, promoting mitochondrial outer membrane permeabilization and the final activation of the executioner caspase cascade.

When persistent viral infection leads to insurmountable ER stress, the UPR transitions from a cytoprotective to a lethal signal, initiating PCD to eliminate the compromised cell. In animal cells, this primarily manifests as apoptosis, a highly regulated process orchestrated by the convergence of all three UPR sensors (117, 118). Figure 5 provides a schematic overview of these pro-apoptotic pathways. Under prolonged stress, the PERK arm, *via* eIF2α phosphorylation, selectively translates the transcription factor ATF4, which in turn induces C/EBP homologous protein (CHOP). As shown in Figure 5, CHOP acts as a central executioner, shifting the cellular balance toward death by upregulating pro-apoptotic Bcl-2 family members and downregulating anti-apoptotic ones, leading to Mitochondrial outer membrane permeabilization (MOMP) and subsequent caspase activation (119, 120, 121) (Fig. 5). Concurrently, the IRE1α sensor, under severe stress, recruits the adaptor protein TRAF2 to activate the ASK1-JNK cascade, which promotes apoptosis by phosphorylating Bcl-2 family proteins. While the ATF6 pathway is primarily adaptive, it can contribute to apoptosis by amplifying signals from the other two branches (118, 122) (Fig. 5). This network, culminating in the activation of caspases-12, -9, and -3, represents the host's ultimate strategy to prevent viral propagation (Fig. 5).

Viruses, in turn, have evolved sophisticated mechanisms to manipulate these precise signaling nodes, hijacking the host's self-destruct program to serve their own lifecycle (Fig. 5). The targets vary significantly. Some viruses focus on the CHOP-dependent pathway. JeV, for instance, induces a broad UPR activation that culminates in a significant elevation of CHOP, driving cells into apoptosis (123) (Fig. 5). HCV provides a more intricate example: expression of its core protein depletes ER calcium stores, inducing CHOP-mediated apoptosis linked to chronic liver disease. Notably, since HCV actively inhibits the PERK pathway, this effect is mediated by the ATF6 and IRE1α-JNK pathways (124, 125). Other viruses directly engage specific caspase cascades. Bovine viral diarrhea virus (BVDV), a positive-sense RNA virus, specifically leverages the PERK-eIF2α axis to hyperphosphorylate eIF2α and activate caspase-12, a process central to its pathogenicity (126). ASFV exploits the ATF6 branch to activate not only caspase-12 but also the downstream executioner caspases-3 and -9, inducing a robust apoptosis thought to facilitate viral release (86) (Fig. 5). The causal link between UPR and apoptosis is further cemented by Coxsackievirus A16 (CVA16), where infection increases caspase-3, -8, and -9-dependent apoptosis, an effect that can be fully mitigated by a chemical chaperone that reduces the initial ER stress (127).

When the ER undergoes excessive stress due to the accumulation of unfolded or misfolded proteins (caused by factors such as hypoxia, nutrient deficiency, or viral infections) the UPR is activated to attempt to restore ER homeostasis (117). However, if this stress persists for too long or becomes too intense, the UPR shifts from a protective mechanism to a pro-apoptotic signal, inducing PCD (primarily apoptosis) to eliminate damaged cells and safeguard the organism, a process orchestrated by the synergistic actions of three main UPR sensors (PERK, IRE1, and ATF6), each playing distinct yet complementary roles that ultimately converge on the mitochondrial-mediated apoptosis pathway (118).

Host–virus conflict over cellular fate is mirrored in plant systems, where the UPR is tightly linked to the hypersensitive response (HR), a form of PCD central to plant immunity. Here, the life-or-death decision is also governed by a balance of UPR signals. The IRE1-bZIP60 pathway and the bZIP28 pathway jointly regulate the expression of the key pro-death transcription factor NAC089, which can trigger PCD by activating downstream effectors like metacaspases (30, 128, 129, 130). The central role of this axis in antiviral defense is highlighted by the fact that bZIP60 homologs are induced during viral infections and are critical for host resistance (131, 132, 133, 134). This delicate balance provides clear targets for viral manipulation, demonstrating how tipping this balance can drastically alter the cell's fate (135). Thus, whether in animals or plants, viruses act as skilled puppet masters that pull specific strings within the UPR to precisely control the host cell's demise.

## Viruses modulate the UPR to balance cell death and robust infection

The ER is the central hub for viral replication. While the initial phases of the UPR can be beneficial, expanding the very membrane and protein-folding capacity a virus needs, a sustained response is a death sentence, designed to shut down translation and trigger apoptosis. Therefore, to establish a robust infection, viruses must do more than simply block the UPR; they must precisely remodel it. This section delves into the molecular tactics that viruses employ to resolve this conflict.

### Strategies in animal viruses

**Figure 6:**
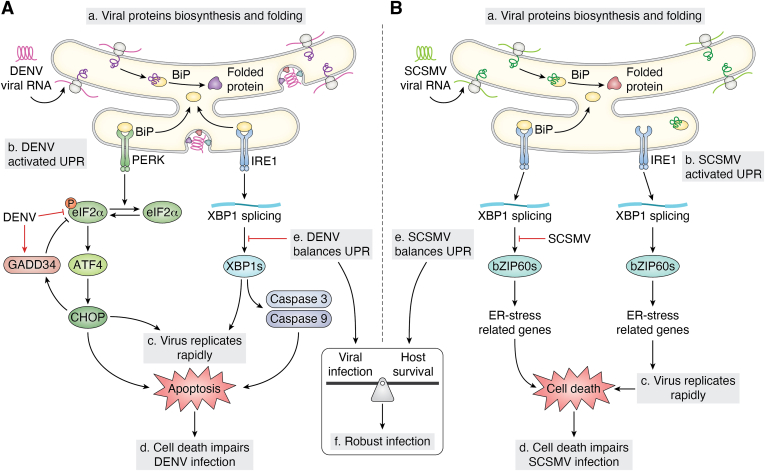
**Viruses modulate the UPR to balance cell death and robust infection**. The *red arrows* in this figure highlight the viral strategies employed to balance the UPR. Effective UPR modulation is critical for preventing premature host cell death and facilitating efficient viral replication, ultimately leading to robust infection. *A*, DENV infection in mammalian cells. *a*, high levels of viral protein synthesis and folding within the ER induce significant ER stress. *b*, this stress activates all three UPR sensors (PERK, IRE1, and ATF6). While the UPR initially triggers host defense mechanisms such as eIF2α-mediated translational arrest (*via* PERK) and activation of pro-apoptotic pathways (*via* PERK and IRE1), (*e*) DENV actively balances the UPR to promote (*c*) rapid viral replication and (*f*) robust infection while maintaining host cell viability. DENV achieves this by upregulating GADD34 to dephosphorylate eIF2α, relieving translational inhibition. Crucially, DENV decouples the IRE1 pathway, suppressing its pro-apoptotic arm (Caspase 3/9 activation) while preserving the pro-survival XBP1s-mediated ER expansion necessary for viral replication complexes (VRCs). This delicate balance prevents premature (*d*) apoptosis, ensuring the host cell remains a viable platform for viral production and thus promoting (*f*) robust infection and host survival long enough for efficient viral propagation. *B*, SCSMV infection in plant cells. A functionally analogous strategy is observed in plants. *a*, SCSMV protein synthesis in the ER induces stress, (*b*) activating the IRE1-bZIP60 UPR pathway, which typically has pro-survival functions in plants. *e*, SCSMV employs its P1 protein to balance the UPR by directly inhibiting IRE1-mediated *bZIP60* mRNA splicing. This targeted inhibition dampens host defense responses that could clear the virus. However, this inhibition must be carefully calibrated. Excessive inhibition could lead to overwhelming ER stress and premature (*d*) host cell death, thus hindering infection. By maintaining an optimal level of UPR activation, SCSMV avoids triggering strong host defenses or causing excessive cellular damage, thereby supporting (*c*) viral replication and (*f*) robust infection and prolonging host cell survival to facilitate systemic spread. In both animal and plant systems, the ability of viruses to precisely modulate the UPR (preventing excessive activation or suppression) is a key determinant of successful infection and host survival long enough for effective viral propagation. The *red arrows* in the figure highlight these critical balancing acts undertaken by the viruses.

Infection by both DENV and HCV is characterized by the activation of all three UPR sensors, a finding consistent across both patient samples and cell culture models (79, 136). Figure 6*A* provides a visual schematic of this process for flaviviruses like DENV. The infection cascade begins with the massive synthesis and folding of viral proteins, which inevitably overwhelms the ER's capacity and induces ER stress (Fig. 6*A*, panel a). To maintain cellular homeostasis, the host initiates the UPR (Fig. 6*A*, panel b). However, viruses have evolved strategies to exploit the UPR; specifically, activating the IRE1 pathway facilitates ER membrane expansion and the expression of chaperones, which further aids in the folding and replication of viral proteins (Fig. 6*A*, panel c). Subsequently, an over-activated UPR leads to a terminal "defensive" response, aiming to eliminate the infected cell *via* apoptosis. However, despite confronting this host response, these two closely related flaviviruses have evolved distinct subversive strategies.

For DENV, the regulation of the UPR exhibits significant complexity and distinct temporal kinetics. At this critical juncture, the virus must intervene by performing a precise balancing act to modulate the UPR, a concept highlighted by the red arrows in Figure 6*A* (panel e). Early activation of the PERK pathway phosphorylates eIF2α, leading to a host translational arrest, which is an effective antiviral mechanism. However, DENV rapidly reverses this phosphorylation, presumably by upregulating the host phosphatase GADD34, thereby lifting the translational blockade to ensure continuous and efficient synthesis of viral proteins (137, 138). Concurrently, DENV actively co-opts the IRE1-XBP1 pathway. Multiple viral proteins (prM, E, NS1, NS2A, NS2B, NS4B) activate IRE1, leading to the splicing of *XBP1* (139). The activated XBP1s not only alleviates ER stress by activating the ERAD pathway but, more importantly, drives the massive expansion of ER membranes, providing the physical platform for the formation of viral replication complexes (VRCs) (139).

In contrast, HCV appears to favor a strategy of evasion over DENV's active reversal when facing PERK-mediated translational arrest. Studies suggest that the HCV internal ribosome entry site (IRES) provides it with intrinsic flexibility, allowing it to switch between eIF2α-dependent and -independent translation modes based on the cellular phosphorylation status of eIF2α (140, 141, 142). This dynamic adaptability is likely key to HCV's ability to establish persistent, long-term infections.

Despite these strategic differences, both DENV and HCV must solve a common puzzle: how to harness the pro-survival benefits of the IRE1 pathway (XBP1s-mediated effects) while evading its pro-death consequences (JNK-mediated apoptosis). By successfully executing this strategy, the virus prevents premature host cell apoptosis (Fig. 6*A*, panel d), which would otherwise terminate the infection. Evidence suggests that DENV can suppress apoptotic mediators downstream of the IRE1-XBP1 pathway, thereby enhancing the survival of infected cells while simultaneously exploiting the pathway for its own replication (138, 139). These signalling changes ensures the host cell remains a viable factory, allowing the virus to replicate rapidly. A key experiment supports this: in DENV-infected cells, siRNA-mediated knockdown of *XBP1* significantly increased the cytopathic effect and elevated levels of pro-caspase 3, an apoptosis marker (139). The cumulative result of this finely tuned manipulation is a robust infection (Fig. 6*A*, panel f), where viral progeny is maximized without killing the host too quickly. This observation inversely demonstrates that an intact IRE1-XBP1 pathway plays a crucial cytoprotective and anti-apoptotic role during DENV infection. In this manner, viruses perform a precise decoupling of the IRE1α signaling pathway, selectively silencing its detrimental pro-apoptotic arm while preserving the pro-survival branches that provide resources and space for viral replication. This ability to uncouple the pro-survival from the pro-death functions of IRE1 is a cornerstone of flavivirus strategy, ensuring that the host cell viral factory remains operational for an extended period to maximize viral progeny. Interestingly, even within flaviviruses, HCV exhibits a further layer of refined control. Studies show that while HCV infection enhances *XBP1* mRNA splicing, it limits the transcription of XBP1s target genes, a move that may facilitate the accumulation of viral glycoproteins and reduce the production of XBP1-mediated pro-inflammatory factors (143, 144).

The strategy of fine-tuned modulation by flaviviruses stands in stark contrast to the “direct confrontation” approach employed by other viral families. On the PERK pathway, many viruses launch multi-pronged attacks to forcibly lift translational inhibition. For example, the γ34.5 protein of herpes simplex virus (HSV), a host GADD34 homolog, recruits the cellular phosphatase PP1A to directly dephosphorylate eIF2α (145, 146), while its gB glycoprotein can directly bind and inhibit PERK phosphorylation (147). Adenovirus (ADV) utilizes its virus-associated RNA 1 (VA RNA1) as a PKR inhibitor and employs the E1B-55 K/E4orf6 protein complex to inhibit eIF2α phosphorylation during late infection, thereby dismantling the host's translational shutdown mechanism (148, 149). This strategic divergence is even more pronounced on the IRE1 pathway. While flaviviruses carefully perform this ‘decoupling,' other viruses employ more overwhelming force. The M50 protein of MCMV induces the complete degradation of IRE1, dismantling the pathway at its source (150). HSV uses its UL41 protein as a specific ribonuclease inhibitor to directly block IRE1's enzymatic activity (150).

In essence, the many examples of viral alterations illustrated in this review illustrate a fundamental divergence in the evolutionary philosophies of animal viruses for managing the UPR. On one hand, flaviviruses like DENV and HCV act as master regulators, treating the UPR not as a switch to be flipped off, but as a complex system to be sculpted and fine-tuned. On the other hand, viruses like HSV and MCMV adopt a strategy of direct antagonism, employing overwhelming force to degrade or inhibit key UPR components. Whether by meticulous modulation or brute-force suppression, both paths lead to the same critical outcome: balancing the UPR to prevent premature host cell death, thereby ensuring a robust infection.

### Strategies in plant viruses

In the plant kingdom, the arms race between viruses and the host UPR is equally intense, revealing a parallel evolutionary logic to that of animal viruses. Figure 6*B* illustrates this compelling case of convergent evolution. The plant UPR primarily consists of two branches: the IRE1-bZIP60 axis and the bZIP17/28 axis. Upon viral infection and subsequent ER stress, both pathways can be activated to regulate downstream pro-survival or pro-death genes, critically influencing the fate of viral replication (116). The induction of ER stress stems from the drastic remodeling of host membrane systems by viruses to construct their "replication factories." For instance, in the *Potyviridae* family, the integral membrane protein 6K2 is a key architect of this process. After anchoring to the ER, 6K2 hijacks the host COPII secretory pathway, inducing complex ER reorganization to form vesicles that enclose the viral replication complexes (VRCs) (50, 151). This profound disruption of ER homeostasis inevitably triggers ER stress (Fig. 6*B*, panel a), and to maintain cellular homeostasis, the host activates the UPR (Fig. 6*B*, panel b) sounding a "survival alarm" for the virus.

To counter this alarm, different plant viruses have evolved diverse regulatory strategies to counter the host UPR. For example, sugarcane streak mosaic virus (SCSMV) employs its P1 protein to bind a key stem-loop structure in *bZIP60* mRNA, directly preventing its processing by IRE1 (152). This action is a clear example of the viral balancing act highlighted by the red arrow in Figure 6*B* (panel e). However, because the IRE1-bZIP60 pathway functions in a pro-survival capacity in plants, this inhibition by P1 is a double-edged sword. Excessive inhibition can disrupt cellular homeostasis and lead to PCD (Fig. 6*B*, panel d). Therefore, the survival of SCSMV depends on maintaining this inhibition at a specific level that both dampens host defenses and avoids premature death of the host cell. Maintaining host cell viability is crucial as it provides a stable environment for the virus to replicate (Fig. 6*B*, panel c). Ultimately, this successful modulation secures a stable and systemic infection, culminating in a robust infection (Fig. 6*B*, panel f) throughout the host plant.

Other viruses have adopted different approaches, involving the active induction and subsequent modulation of the UPR. The NSvc2 and NSvc4 proteins of rice stripe virus (RSV) specifically activate the bZIP17/28 branch of the UPR, which in turn induces host autophagy to degrade viral proteins (153, 154). Concurrently, the virus utilizes host type I J-domain proteins (NbMIP1s) to protect its proteins from excessive degradation (154). This series of interactions forms a regulatory loop that maintains viral replication at a level tolerable to the host. A similar logic is evident in tomato yellow leaf curl China virus (TYLCCNV). Its βC1 protein exports the activated bZIP60 transcription factor from the nucleus *via* the exportin 1 (XPO1) pathway (155), while its βV1 protein induces the bZIP17/28 pathway and activates autophagy to partially degrade viral proteins (156). This "self-limiting" regulation of viral proteins, by actively slowing its own replication rate, effectively circumvents intense ER stress and host cell death, thereby creating favorable conditions for stable and persistent systemic infection.

The virus products that alter the UPR in the plant kingdom provide a compelling narrative of convergent evolution. The strategic approaches observed in animal viruses are functionally mirrored in plants. The direct inhibition of bZIP60 processing by SCSMV, for instance, represents a strategy of targeted pathway antagonism, analogous to the methods used by viruses like HSV or MCMV to inactivate key UPR components. Concurrently, the self-limiting feedback loops employed by RSV and TYLCCNV reflect a different approach centered on dynamic modulation, which parallels the regulatory principles seen with flaviviruses. Ultimately, whether in a plant or an animal host, the underlying evolutionary logic is identical: the virus must precisely balance the UPR to prevent premature cell death, thereby securing a stable and systemic infection.

## Evolving interplay between viruses and host UPR

This review presents the intricate interplay between viruses and the host UPR, revealing how viruses exploit, fine-tune, and evade this conserved cellular stress response to facilitate infection. By selectively modulating the IRE1, PERK, and ATF6 signaling pathways, viruses enhance protein folding, restructure the ER to support replication, and evade host immune surveillance. However, the dual nature of UPR presents a challenge: while moderate activation is advantageous for viral propagation, excessive or prolonged stress can trigger apoptosis, leading to premature host cell death and restricting viral replication. This delicate balance highlights the evolutionary arms race between the host defense and viral adaptation, where viruses must optimize UPR activation to maximize replication without inducing fatal levels of cellular stress.

Despite significant progress, our understanding of viral control over the UPR remains incomplete. In mammalian systems, direct manipulation of UPR sensors and downstream effectors is increasingly well-characterized, yet whether analogous mechanisms operate in plants is far less clear. Existing evidence is fragmented: TuMV 6K2 promotes replication complex formation through IRE1/bZIP60, SCSMV P1 interferes with bZIP60 splicing, and TYLCCNV βC1 redirects bZIP60 to the cytoplasm while inducing autophagy. Together, these observations raise a critical unresolved question of whether plant viruses directly regulate UPR sensors such as bZIP17/28 or bZIP60 to suppress UPR-mediated PCD signaling, and how such regulation intersects with plant-specific PCD pathways. Equally enigmatic is the broader role of the UPR in shaping lipid metabolism. Viral infections remodel ER membranes and alter lipid dynamics, yet the causal links between these metabolic shifts, UPR sensor activation, and viral replication remain poorly defined. Understanding whether viruses co-opt lipid regulators such as sterol regulatory element-binding protein (SREBP) or peroxisome proliferator-activated receptor (PPAR) to sustain membrane expansion will be key to resolving this interface.

Exploring those intersections will provide valuable insights into novel antiviral targets. The selective modulation of UPR components through chemical genetics and small-molecule inhibitors holds promise for therapeutic intervention, not only in viral infections but also in nonviral diseases exacerbated by chronic UPR dysregulation. By integrating virology, structural biology, and metabolic research, future studies will pave the way for innovative strategies to manipulate UPR dynamics, providing new avenues for both antiviral therapies and broader biomedical applications.

## Conflict of interest

The authors declare that they do not have any conflicts of interest with the content of this article.

## References

[1] Schwarz D.S., Blower M.D. (2016). The endoplasmic reticulum: structure, function and response to cellular signaling. Cell. Mol. Life Sci..

[2] van der Zand A., Gent J., Braakman I., Tabak H.F. (2012). Biochemically distinct vesicles from the endoplasmic reticulum fuse to form peroxisomes. Cell.

[3] Evans E.A., Gilmore R., Blobel G. (1986). Purification of microsomal signal peptidase as a complex. Proc. Natl. Acad. Sci. U. S. A..

[4] Blobel G. (1980). Intracellular protein topogenesis. Proc. Natl. Acad. Sci. U. S. A..

[5] Wang M., Kaufman R.J. (2016). Protein misfolding in the endoplasmic reticulum as a conduit to human disease. Nature.

[6] Itskanov S., Wang L., Junne T., Sherriff R., Xiao L., Blanchard N. (2023). A common mechanism of Sec61 translocon inhibition by small molecules. Nat. Chem. Biol..

[7] Park E., Rapoport T.A. (2012). Mechanisms of Sec61/SecY-Mediated protein translocation across membranes. Annu. Rev. Biophys..

[8] Jiang Y., Tao Z., Chen H., Xia S. (2021). Endoplasmic reticulum quality control in immune cells. Front. Cell. Dev. Biol.

[9] Vembar S.S., Brodsky J.L. (2008). One step at a time: endoplasmic reticulum-associated degradation. Nat. Rev. Mol. Cell Biol..

[10] Kaufman R.J. (2002). Orchestrating the unfolded protein response in health and disease. J. Clin. Invest.

[11] Howell S.H. (2013). Endoplasmic reticulum stress responses in plants. Annu. Rev. Plant Biol..

[12] Vitale A., Boston R.S. (2008). Endoplasmic reticulum quality control and the unfolded protein response: insights from plants. Traffic.

[13] Ravindran M.S., Bagchi P., Cunningham C.N., Tsai B. (2016). Opportunistic intruders: how viruses orchestrate ER functions to infect cells. Nat. Rev. Microbiol..

[14] Chavan M., Lennarz W. (2006). The molecular basis of coupling of translocation and N-glycosylation. Trends Biochem. Sci..

[15] Bulleid N.J. (2012). Disulfide bond formation in the mammalian endoplasmic reticulum. Cold Spring Harb. Perspect. Biol..

[16] Behnke J., Feige M.J., Hendershot L.M. (2015). BiP and its nucleotide exchange factors Grp170 and Sil1: mechanisms of action and biological functions. J. Mol. Biol..

[17] Miller E.A., Schekman R. (2013). COPII - a flexible vesicle formation system. Curr. Opin. Cell Biol.

[18] Lam A.K., Galione A. (2013). The endoplasmic reticulum and junctional membrane communication during calcium signaling. Biochim. Biophys. Acta.

[19] Tannous A., Pisoni G.B., Hebert D.N., Molinari M. (2015). N-linked sugar-regulated protein folding and quality control in the ER. Semin. Cell Dev. Biol..

[20] Natter K., Leitner P., Faschinger A., Wolinski H., McCraith S., Fields S. (2005). The spatial organization of lipid synthesis in the yeast Saccharomyces cerevisiae derived from large scale green fluorescent protein tagging and high resolution microscopy. Mol. Cell Proteomics..

[21] Saptarshi N., Porter L.F., Paraoan L. (2022). PERK/EIF2AK3 integrates endoplasmic reticulum stress-induced apoptosis, oxidative stress and autophagy responses in immortalised retinal pigment epithelial cells. Sci. Rep..

[22] Zhang L., Wang A., Wang A., Zhou X. (2016). Current Research Topics in Plant Virology.

[23] Kim J.-S., Mochida K., Shinozaki K. (2022). ER stress and the unfolded protein response: homeostatic regulation coordinate plant survival and growth. Plants.

[24] Ron D., Walter P. (2007). Signal integration in the endoplasmic reticulum unfolded protein response. Nat. Rev. Mol. Cell Biol..

[25] Hetz C., Martinon F., Rodriguez D., Glimcher L.H. (2011). The unfolded protein response: integrating stress signals through the stress sensor IRE1α. Physiol. Rev..

[26] Afrin T., Diwan D., Sahawneh K., Pajerowska-Mukhtar K. (2019). Multilevel regulation of endoplasmic reticulum stress responses in plants: where old roads and new paths meet. J. Exp. Bot..

[27] Korennykh A.V., Egea P.F., Korostelev A.A., Finer-Moore J., Zhang C., Shokat K.M. (2009). The unfolded protein response signals through high-order assembly of Ire1. Nature.

[28] Yoshida H., Matsui T., Yamamoto A., Okada T., Mori K. (2001). *XBP1* mRNA is induced by ATF6 and spliced by IRE1 in response to ER stress to produce a highly active transcription factor. Cell.

[29] Grootjans J., Kaser A., Kaufman R.J., Blumberg R.S. (2016). The unfolded protein response in immunity and inflammation. Nat. Rev. Immunol..

[30] Deng Y., Humbert S., Liu J.X., Srivastava R., Rothstein S.J., Howell S.H. (2011). Heat induces the splicing by IRE1 of a mRNA encoding a transcription factor involved in the unfolded protein response in *Arabidopsis*. Proc. Natl. Acad. Sci. U. S. A..

[31] Nagashima Y., Mishiba K., Suzuki E., Shimada Y., Iwata Y., Koizumi N. (2011). *Arabidopsis* IRE1 catalyses unconventional splicing of *bZIP60* mRNA to produce the active transcription factor. Sci. Rep..

[32] Hollien J., Weissman J.S. (2006). Decay of endoplasmic reticulum-localized mRNAs during the unfolded protein response. Science.

[33] Lin J.H., Li H., Yasumura D., Cohen H.R., Zhang C., Panning B. (2007). IRE1 signaling affects cell fate during the unfolded protein response. Science.

[34] Rutkowski D.T., Arnold S.M., Miller C.N., Wu J., Li J., Gunnison K.M. (2006). Adaptation to ER stress is mediated by differential stabilities of pro-survival and pro-apoptotic mRNAs and proteins. PLoS Biol..

[35] Ghosh R., Wang L., Wang E.S., Perera B.G., Igbaria A., Morita S. (2014). Allosteric inhibition of the IRE1α RNase preserves cell viability and function during endoplasmic reticulum stress. Cell.

[36] Lerner A.G., Upton J.P., Praveen P.V., Ghosh R., Nakagawa Y., Igbaria A. (2012). IRE1α induces thioredoxin-interacting protein to activate the NLRP3 inflammasome and promote programmed cell death under irremediable ER stress. Cell Metab..

[37] Bertolotti A., Zhang Y., Hendershot L.M., Harding H.P., Ron D. (2000). Dynamic interaction of BiP and ER stress transducers in the unfolded-protein response. Nat. Cell Biol..

[38] Cui W., Li J., Ron D., Sha B. (2011). The structure of the PERK kinase domain suggests the mechanism for its activation. Acta Crystallogr. D Biol. Crystallogr..

[39] Harding H.P., Zhang Y., Zeng H., Novoa I., Lu P.D., Calfon M. (2003). An integrated stress response regulates amino acid metabolism and resistance to oxidative stress. Mol. Cell.

[40] Lo S.C., Li X., Henzl M.T., Beamer L.J., Hannink M. (2006). Structure of the Keap1:Nrf2 interface provides mechanistic insight into Nrf2 signaling. EMBO J.

[41] Lin J.H., Li H., Zhang Y., Ron D., Walter P. (2009). Divergent effects of PERK and IRE1 signaling on cell viability. PLoS One.

[42] Mounir Z., Krishnamoorthy J.L., Wang S., Papadopoulou B., Campbell S., Muller W.J. (2011). Akt determines cell fate through inhibition of the PERK-eIF2α phosphorylation pathway. Sci. Signal..

[43] Haze K., Yoshida H., Yanagi H., Yura T., Mori K. (1999). Mammalian transcription factor ATF6 is synthesized as a transmembrane protein and activated by proteolysis in response to endoplasmic reticulum stress. Mol. Biol. Cell.

[44] Ye J., Rawson R.B., Komuro R., Chen X., Davé U.P., Prywes R. (2000). ER stress induces cleavage of membrane-bound ATF6 by the same proteases that process SREBPs. Mol. Cell.

[45] Hetz C., Zhang K., Kaufman R.J. (2020). Mechanisms, regulation and functions of the unfolded protein response. Nat. Rev. Mol. Cell Biol..

[46] Liu J.X., Srivastava R., Che P., Howell S.H. (2007). An endoplasmic reticulum stress response in *Arabidopsis* is mediated by proteolytic processing and nuclear relocation of a membrane-associated transcription factor, bZIP28. Plant Cell.

[47] Srivastava R., Deng Y., Shah S., Rao A.G., Howell S.H. (2013). BINDING PROTEIN is a master regulator of the endoplasmic reticulum stress sensor/transducer bZIP28 in *Arabidopsis*. Plant Cell.

[48] Restrepo-Hartwig M., Ahlquist P. (1999). Brome mosaic virus RNA replication proteins 1a and 2a colocalize and 1a independently localizes on the yeast endoplasmic reticulum. J. Virol..

[49] Ju H.-J., Samuels T.D., Wang Y.-S., Blancaflor E., Payton M., Mitra R. (2005). The potato virus X TGBp2 movement protein associates with endoplasmic reticulum-derived vesicles during virus infection. Plant Physiol..

[50] Schaad M.C., Jensen P.E., Carrington J.C. (1997). Formation of plant RNA virus replication complexes on membranes: role of an endoplasmic reticulum-targeted viral protein. EMBO J..

[51] Carette J.E., Stuiver M., Lent J.V., Wellink J., Kammen A.V. (2000). Cowpea mosaic virus infection induces a massive proliferation of endoplasmic reticulum but not golgi membranes and is dependent on de novo membrane synthesis. J. Virol..

[52] McCartney A.W., Greenwood J.S., Fabian M.R., White K.A., Mullen R.T. (2005). Localization of the tomato bushy stunt virus replication protein p33 reveals a peroxisome-to-endoplasmic reticulum sorting pathway. Plant Cell.

[53] Monel B., Compton A.A., Bruel T., Amraoui S., Burlaud-Gaillard J., Roy N. (2017). Zika virus induces massive cytoplasmic vacuolization and paraptosis-like death in infected cells. EMBO J.

[54] Paul D., Bartenschlager R. (2015). *Flaviviridae* replication organelles: oh, what a tangled web we weave. Annu. Rev. Virol..

[55] den Boon J.A., Ahlquist P. (2010). Organelle-like membrane compartmentalization of positive-strand RNA virus replication factories. Annu. Rev. Microbiol..

[56] Pegg C.E., Zaichick S.V., Bomba-Warczak E., Jovasevic V., Kim D., Kharkwal H. (2021). Herpesviruses assimilate kinesin to produce motorized viral particles. Nature.

[57] Verchot J. (2011). Wrapping membranes around plant virus infection. Curr. Opin. Virol..

[58] Kamauchi S., Nakatani H., Nakano C., Urade R. (2005). Gene expression in response to endoplasmic reticulum stress in *Arabidopsis thaliana*. FEBS J..

[59] Martínez I.M., Chrispeels M.J. (2003). Genomic analysis of the unfolded protein response in *Arabidopsis* shows its connection to important cellular processes. Plant Cell.

[60] Pollier J., Moses T., González-Guzmán M., De Geyter N., Lippens S., Bossche R.V. (2013). The protein quality control system manages plant defence compound synthesis. Nature.

[61] Hetz C. (2012). The unfolded protein response: controlling cell fate decisions under ER stress and beyond. Nat. Rev. Mol. Cell Biol..

[62] Welihinda A.A., Kaufman R.J. (1996). The unfolded protein response pathway in saccharomyces cerevisiae: Oligomerization and trans-phosphorylation of Ire1p (Ern1p) are required for kinase activation. J. Biol. Chem..

[63] Wan S., Jiang L. (2016). Endoplasmic reticulum (ER) stress and the unfolded protein response (UPR) in plants. Protoplasma.

[64] Cai Y.M., Yu J., Gallois P. (2014). Endoplasmic reticulum stress-induced PCD and caspase-like activities involved. Front. Plant Sci..

[65] Williams B., Verchot J., Dickman M.B. (2014). When supply does not meet demand-ER stress and plant programmed cell death. Front. Plant Sci..

[66] Echavarría-Consuegra L., Cook G.M., Busnadiego I., Lefèvre C., Keep S., Brown K. (2021). Manipulation of the unfolded protein response: a pharmacological strategy against coronavirus infection. PLOS Pathog..

[67] Prasad V., Cerikan B., Stahl Y., Kopp K., Magg V., Acosta-Rivero N. (2023). Enhanced SARS-CoV-2 entry via UPR-dependent AMPK-related kinase NUAK2. Mol. Cell.

[68] Marano V., Vlachová Š., Tiano S.M.L., Cortese M. (2024). A portrait of the infected cell: how SARS-CoV-2 infection reshapes cellular processes and pathways. NPJ Viruses.

[69] Tardif K.D., Mori K., Siddiqui A. (2002). Hepatitis C virus subgenomic replicons induce endoplasmic reticulum stress activating an intracellular signaling pathway. J. Virol..

[70] Lindenbach B.D., Rice C.M. (2013). The ins and outs of hepatitis C virus entry and assembly. Nat. Rev. Microbiol..

[71] Whitham S.A., Quan S., Chang H.S., Cooper B., Estes B., Zhu T. (2003). Diverse RNA viruses elicit the expression of common sets of genes in susceptible *Arabidopsis thaliana* plants. Plant J..

[72] Yang C., Guo R., Jie F., Nettleton D., Peng J., Carr T. (2007). Spatial analysis of *Arabidopsis thaliana* gene expression in response to turnip mosaic virus infection. Mol. Plant Microbe Interact..

[73] Sung S.C., Chao C.Y., Jeng K.S., Yang J.Y., Lai M.M. (2009). The 8ab protein of SARS-CoV is a luminal ER membrane-associated protein and induces the activation of ATF6. Virology.

[74] Watowich S.S., Morimoto R.I., Lamb R.A. (1991). Flux of the paramyxovirus hemagglutinin-neuraminidase glycoprotein through the endoplasmic reticulum activates transcription of the *GRP78-BiP* gene. J. Virol..

[75] Liberman E., Fong Y.L., Selby M.J., Choo Q.L., Cousens L., Houghton M. (1999). Activation of the grp78 and grp94 promoters by hepatitis C virus E2 envelope protein. J. Virol..

[76] Taguwa S., Kambara H., Omori H., Tani H., Abe T., Mori Y. (2009). Cochaperone activity of human butyrate-induced transcript 1 facilitates hepatitis C virus replication through an Hsp90-dependent pathway. J. Virol..

[77] Taguwa S., Okamoto T., Abe T., Mori Y., Suzuki T., Moriishi K. (2008). Human butyrate-induced transcript 1 interacts with hepatitis C virus NS5A and regulates viral replication. J. Virol..

[78] Okamoto T., Nishimura Y., Ichimura T., Suzuki K., Miyamura T., Suzuki T. (2006). Hepatitis C virus RNA replication is regulated by FKBP8 and Hsp90. EMBO J.

[79] Chan S.W. (2014). Unfolded protein response in hepatitis C virus infection. Front. Microbiol..

[80] Bamunusinghe D., Hemenway C.L., Nelson R.S., Sanderfoot A.A., Ye C.M., Silva M.A. (2009). Analysis of potato virus X replicase and TGBp3 subcellular locations. Virology.

[81] Ye C., Dickman M.B., Whitham S.A., Payton M., Verchot J. (2011). The unfolded protein response is triggered by a plant viral movement protein. Plant. Physiol..

[82] Zhang L., Chen H., Brandizzi F., Verchot J., Wang A. (2015). The UPR branch IRE1-bZIP60 in plants plays an essential role in viral infection and is complementary to the only UPR pathway in yeast. PLoS Genet..

[83] Laliberté J.F., Sanfaçon H. (2010). Cellular remodeling during plant virus infection. Annu. Rev. Phytopathol.

[84] Neufeldt C.J., Cortese M., Acosta E.G., Bartenschlager R. (2018). Rewiring cellular networks by members of the *Flaviviridae* family. Nat. Rev. Microbiol..

[85] Chan C.P., Siu K.L., Chin K.T., Yuen K.Y., Zheng B., Jin D.Y. (2006). Modulation of the unfolded protein response by the severe acute respiratory syndrome coronavirus spike protein. J. Virol..

[86] Galindo I., Hernáez B., Muñoz-Moreno R., Cuesta-Geijo M.A., Dalmau-Mena I., Alonso C. (2012). The ATF6 branch of unfolded protein response and apoptosis are activated to promote African swine fever virus infection. Cell Death Dis..

[87] Prasad V., Suomalainen M., Jasiqi Y., Hemmi S., Hearing P., Hosie L. (2020). The UPR sensor IRE1α and the adenovirus E3-19K glycoprotein sustain persistent and lytic infections. Nat. Commun..

[88] Hinte F., van Anken E., Tirosh B., Brune W. (2020). Repression of viral gene expression and replication by the unfolded protein response effector XBP1u. Elife.

[89] Olzmann J.A., Kopito R.R., Christianson J.C. (2013). The mammalian endoplasmic reticulum-associated degradation system. Cold Spring Harb Perspect. Biol..

[90] Kruse K.B., Brodsky J.L., McCracken A.A. (2006). Autophagy: an ER protein quality control process. Autophagy.

[91] Boomen D.J.H., Lehner P.J. (2015). Identifying the ERAD ubiquitin E3 ligases for viral and cellular targeting of MHC class I. Mol. Immunol..

[92] Wiertz E.J., Jones T.R., Sun L., Bogyo M., Geuze H.J., Ploegh H.L. (1996). The human cytomegalovirus US11 gene product dislocates MHC class I heavy chains from the endoplasmic reticulum to the cytosol. Cell.

[93] Magadan J.G., Perez-Victoria F.J., Sougrat R., Ye Y., Strebel K., Bonifacino J.S. (2010). Multilayered mechanism of CD4 downregulation by HIV-1 Vpu involving distinct ER retention and ERAD targeting steps. PLoS Pathog..

[94] Tabata K., Arakawa M., Ishida K., Kobayashi M., Nara A., Sugimoto T. (2021). Endoplasmic reticulum-associated degradation controls virus protein homeostasis, which is required for flavivirus propagation. J. Virol..

[95] Lazar C., Macovei A., Petrescu S., Branza-Nichita N. (2012). Activation of ERAD pathway by human hepatitis B virus modulates viral and subviral particle production. PLoS One.

[96] Saeed M., Suzuki R., Watanabe N., Masaki T., Tomonaga M., Muhammad A. (2011). Role of the endoplasmic reticulum-associated degradation (ERAD) pathway in degradation of hepatitis C virus envelope proteins and production of virus particles. J. Biol. Chem..

[97] Surjit M., Jameel S., Lal S.K. (2007). Cytoplasmic localization of the ORF2 protein of hepatitis E virus is dependent on its ability to undergo retrotranslocation from the endoplasmic reticulum. J. Virol..

[98] Zou L., Wang X., Zhao F., Wu K., Li X., Li Z. (2022). Viruses hijack ERAD to regulate their replication and propagation. Int. J. Mol. Sci..

[99] Schelhaas M., Malmström J., Pelkmans L., Haugstetter J., Ellgaard L., Grünewald K. (2007). Simian virus 40 depends on ER protein folding and quality control factors for entry into host cells. Cell.

[100] Geiger R., Andritschke D., Friebe S., Herzog F., Luisoni S., Heger T. (2011). BAP31 and BiP are essential for dislocation of SV40 from the endoplasmic reticulum to the cytosol. Nat. Cell Biol..

[101] Bennett S.M., Jiang M., Imperiale M.J. (2013). Role of cell-type-specific endoplasmic reticulum-associated degradation in polyomavirus trafficking. J. Virol..

[102] Levine B., Klionsky D.J. (2004). Development by self-digestion: molecular mechanisms and biological functions of autophagy. Dev. Cell.

[103] Shintani T., Klionsky D.J. (2004). Autophagy in health and disease: a double-edged sword. Science.

[104] Qi Z., Chen L. (2019). Endoplasmic reticulum stress and autophagy. Adv. Exp. Med. Biol..

[105] Salazar M., Carracedo A., Salanueva I.J., Hernández-Tiedra S., Lorente M., Egia A. (2009). Cannabinoid action induces autophagy-mediated cell death through stimulation of ER stress in human glioma cells. J. Clin. Invest..

[106] Song S., Tan J., Miao Y., Li M., Zhang Q. (2017). Crosstalk of autophagy and apoptosis: involvement of the dual role of autophagy under ER stress. J. Cell Physiol..

[107] Liu Y., Burgos J.S., Deng Y., Srivastava R., Howell S.H., Bassham D.C. (2012). Degradation of the endoplasmic reticulum by autophagy during endoplasmic reticulum stress in *Arabidopsis*. Plant Cell.

[108] Hafrén A., Macia J.L., Love A.J., Milner J.J., Drucker M., Hofius D. (2017). Selective autophagy limits cauliflower mosaic virus infection by NBR1-mediated targeting of viral capsid protein and particles. Proc. Natl. Acad. Sci. U. S. A..

[109] Ke P.Y., Chen S.S. (2014). Autophagy in hepatitis C virus-host interactions: potential roles and therapeutic targets for liver-associated diseases. World J. Gastroenterol..

[110] Ke P.Y., Chen S.S. (2011). Activation of the unfolded protein response and autophagy after hepatitis C virus infection suppresses innate antiviral immunity *in vitro*. J. Clin. Invest..

[111] Huang H., Kang R., Wang J., Luo G., Yang W., Zhao Z. (2013). Hepatitis C virus inhibits AKT-tuberous sclerosis complex (TSC), the mechanistic target of rapamycin (MTOR) pathway, through endoplasmic reticulum stress to induce autophagy. Autophagy.

[112] Jordan T.X., Randall G. (2012). Manipulation or capitulation: virus interactions with autophagy. Microb. Infect..

[113] Marquez R.T., Xu L. (2012). Bcl-2:Beclin 1 complex: multiple, mechanisms regulating autophagy/apoptosis toggle switch. Am. J. Cancer Res..

[114] Lee Y.R., Kuo S.H., Lin C.Y., Fu P.J., Lin Y.S., Yeh T.M. (2018). Dengue virus-induced ER stress is required for autophagy activation, viral replication, and pathogenesis both *in vitro* and *in vivo*. Sci. Rep..

[115] Ranjitha H.B., Ammanathan V., Guleria N., Hosamani M., Sreenivasa B.P., Dhanesh V.V. (2020). Foot-and-mouth disease virus induces PERK-mediated autophagy to suppress the antiviral interferon response. J. Cell Sci..

[116] Li F., Zhang C., Tang Z., Zhang L., Dai Z., Lyu S. (2020). A plant RNA virus activates selective autophagy in a UPR-dependent manner to promote virus infection. New Phytol..

[117] Shore G.C., Papa F.R., Oakes S.A. (2011). Signaling cell death from the endoplasmic reticulum stress response. Curr. Opin. Cell Biol..

[118] Hetz C., Papa F.R. (2018). The unfolded protein response and cell fate control. Mol. Cell.

[119] Urra H., Dufey E., Lisbona F., Rojas-Rivera D., Hetz C. (2013). When ER stress reaches a dead end. Biochim. Biophys. Acta.

[120] Upton J.P., Wang L., Han D., Wang E.S., Huskey N.E., Lim L. (2012). IRE1α cleaves select microRNAs during ER stress to derepress translation of proapoptotic Caspase-2. Science.

[121] Hiramatsu N., Chiang W.C., Kurt T.D., Sigurdson C.J., Lin J.H. (2015). Multiple mechanisms of unfolded protein response-induced cell death. Am. J. Pathol..

[122] Fribley A., Zhang K., Kaufman R.J. (2009). Regulation of apoptosis by the unfolded protein response. Methods Mol. Biol..

[123] Su H.L., Liao C.L., Lin Y.L. (2002). Japanese encephalitis virus infection initiates endoplasmic reticulum stress and an unfolded protein response. J. Virol..

[124] Benali-Furet N.L., Chami M., Houel L., De Giorgi F., Vernejoul F., Lagorce D. (2005). Hepatitis C virus core triggers apoptosis in liver cells by inducing ER stress and ER calcium depletion. Oncogene.

[125] Chusri P., Kumthip K., Hong J., Zhu C., Duan X., Jilg N. (2016). HCV induces transforming growth factor β1 through activation of endoplasmic reticulum stress and the unfolded protein response. Sci. Rep..

[126] Jordan R., Wang L., Graczyk T.M., Block T.M., Romano P.R. (2002). Replication of a cytopathic strain of bovine viral diarrhea virus activates PERK and induces endoplasmic reticulum stress-mediated apoptosis of MDBK cells. J. Virol..

[127] Zhu G., Zheng Y., Zhang L., Shi Y., Li W., Liu Z. (2013). Coxsackievirus A16 infection triggers apoptosis in RD cells by inducing ER stress. Biochem. Biophys. Res. Commun..

[128] Iwata Y., Koizumi N. (2005). An *Arabidopsis* transcription factor, AtbZIP60, regulates the endoplasmic reticulum stress response in a manner unique to plants. Proc. Natl. Acad. Sci. U S A..

[129] Lu D.P., Christopher D.A. (2008). Endoplasmic reticulum stress activates the expression of a sub-group of protein disulfide isomerase genes and AtbZIP60 modulates the response in *Arabidopsis thaliana*. Mol. Genet. Genomics.

[130] Yang Z.T., Wang M.J., Sun L., Lu S.J., Bi D.L., Sun L. (2014). The membrane-associated transcription factor NAC089 controls ER-stress-induced programmed cell death in plants. PLoS Genet..

[131] Koizumi N., Martinez I.M., Kimata Y., Kohno K., Sano H., Chrispeels M.J. (2001). Molecular characterization of two *Arabidopsis* Ire1 homologs, endoplasmic reticulum-located transmembrane protein kinases. Plant Physiol..

[132] Takahashi H., Kawakatsu T., Wakasa Y., Hayashi S., Takaiwa F. (2012). A rice transmembrane bZIP transcription factor, OsbZIP39, regulates the endoplasmic reticulum stress response. Plant Cell Physiol..

[133] Hayashi S., Wakasa Y., Takahashi H., Kawakatsu T., Takaiwa F. (2012). Signal transduction by IRE1-mediated splicing of bZIP50 and other stress sensors in the endoplasmic reticulum stress response of rice. Plant J..

[134] Lu S.-J., Yang Z.-T., Sun L., Sun L., Song Z.-T., Liu J.-X. (2012). Conservation of IRE1-Regulated bZIP74 mRNA unconventional splicing in rice (*Oryza sativa* L.) involved in ER stress responses. Mol. Plant.

[135] Xu G., Li S., Xie K., Zhang Q., Wang Y., Tang Y. (2012). Plant ERD2-like proteins function as endoplasmic reticulum luminal protein receptors and participate in programmed cell death during innate immunity. Plant J..

[136] Sessions O.M., Barrows N.J., Souza-Neto J.A., Robinson T.J., Hershey C.L., Rodgers M.A. (2009). Discovery of insect and human dengue virus host factors. Nature.

[137] Umareddy I., Pluquet O., Wang Q.Y., Vasudevan S.G., Chevet E., Gu F. (2007). Dengue virus serotype infection specifies the activation of the unfolded protein response. Virol. J..

[138] Peña J., Harris E. (2011). Dengue virus modulates the unfolded protein response in a time-dependent manner. J. Biol. Chem..

[139] Perera N., Miller J.L., Zitzmann N. (2017). The role of the unfolded protein response in dengue virus pathogenesis. Cell Microbiol.

[140] Khawaja A., Vopalensky V., Pospisek M. (2015). Understanding the potential of hepatitis C virus internal ribosome entry site domains to modulate translation initiation via their structure and function. Wiley Interdiscip. Rev. RNA.

[141] Walsh D., Mohr I. (2011). Viral subversion of the host protein synthesis machinery. Nat. Rev. Microbiol..

[142] Yokoyama T., Machida K., Iwasaki W., Shigeta T., Nishimoto M., Takahashi M. (2019). HCV IRES captures an actively translating 80S ribosome. Mol. Cell.

[143] Tardif K.D., Mori K., Kaufman R.J., Siddiqui A. (2004). Hepatitis C virus suppresses the IRE1-XBP1 pathway of the unfolded protein response. J. Biol. Chem..

[144] Smith J.A. (2014). A new paradigm: innate immune sensing of viruses via the unfolded protein response. Front Microbiol..

[145] Chou J., Chen J.J., Gross M., Roizman B. (1995). Association of a M(r) 90,000 phosphoprotein with protein kinase PKR in cells exhibiting enhanced phosphorylation of translation initiation factor eIF-2 alpha and premature shutoff of protein synthesis after infection with gamma 134.5- mutants of herpes simplex virus 1. Proc. Natl. Acad. Sci. U. S. A..

[146] Spurgeon M.E., Ornelles D.A. (2009). The adenovirus E1B 55-kilodalton and E4 open reading frame 6 proteins limit phosphorylation of eIF2alpha during the late phase of infection. J. Virol..

[147] Mulvey M., Arias C., Mohr I. (2007). Maintenance of endoplasmic reticulum (ER) homeostasis in herpes simplex virus type 1-infected cells through the association of a viral glycoprotein with PERK, a cellular ER stress sensor. J. Virol..

[148] Mathews M.B., Shenk T. (1991). Adenovirus virus-associated RNA and translation control. J. Virol..

[149] Harada J.N., Shevchenko A., Shevchenko A., Pallas D.C., Berk A.J. (2002). Analysis of the adenovirus E1B-55K-anchored proteome reveals its link to ubiquitination machinery. J. Virol..

[150] Stahl S., Burkhart J.M., Hinte F., Tirosh B., Mohr H., Zahedi R.P. (2013). Cytomegalovirus downregulates IRE1 to repress the unfolded protein response. PLoS Pathog..

[151] Jiang J., Patarroyo C., Garcia Cabanillas D., Zheng H., Laliberté J.F. (2015). The vesicle-forming 6K2 protein of turnip mosaic virus interacts with the COPII coatomer Sec24a for viral systemic infection. J. Virol..

[152] Zhang K., Gu T., Xu X., Gan H., Qin L., Feng C. (2023). Sugarcane streak mosaic virus P1 protein inhibits unfolded protein response through direct suppression of *bZIP60U* splicing. PLoS Pathog..

[153] Li C., Zhang T., Liu Y., Li Z., Wang Y., Fu S. (2022). Rice stripe virus activates the bZIP17/28 branch of the unfolded protein response signalling pathway to promote viral infection. Mol. Plant Pathol..

[154] Li C., Xu Y., Fu S., Liu Y., Li Z., Zhang T. (2021). The unfolded protein response plays dual roles in rice stripe virus infection through fine-tuning the movement protein accumulation. PLoS Pathog..

[155] Zhang M., Cao B., Zhang H., Fan Z., Zhou X., Li F. (2023). Geminivirus satellite-encoded βC1 activates UPR, induces bZIP60 nuclear export, and manipulates the expression of bZIP60 downstream genes to benefit virus infection. Sci. China Life Sci..

[156] Hu T., Li C., Liu H., Su C., Wang Y., Li F. (2025). Geminivirus βV1 protein activates bZIP17/28-mediated UPR signaling to facilitate viral pathogenicity but its activity is attenuated by autophagic degradation in plants. Plant Commun..

[157] Medigeshi G.R., Lancaster A.M., Hirsch A.J., Briese T., Lipkin W.I., DeFilippis V. (2007). West Nile virus infection activates the unfolded protein response, leading to CHOP induction and apoptosis. J. Virol..

[158] Sepúlveda-Salinas K.J., Ramos-Castañeda J. (2017). Participation of dengue virus NS4B protein in the modulation of immune effectors dependent on ER stress in insect cells. Cell Stress Chaperones..

[159] Carletti T., Zakaria M.K., Marcello A. (2017). The host cell response to tick-borne encephalitis virus. Biochem. Biophys. Res. Commun..

[160] Turpin J., Frumence E., Harrabi W., Haddad J.G., El Kalamouni C., Desprès P. (2020). Zika virus subversion of chaperone GRP78/BiP expression in A549 cells during UPR activation. Biochimie.

[161] Khongwichit S., Sornjai W., Jitobaom K., Greenwood M., Greenwood M.P., Hitakarun A. (2021). A functional interaction between GRP78 and Zika virus E protein. Sci. Rep..

[162] Mohd Ropidi MI, Khazali A.S., Nor Rashid N., Yusof R. (2020). Endoplasmic reticulum: a focal point of Zika virus infection. J. Biomed. Sci..

[163] He W., Xu H., Gou H., Yuan J., Liao J., Chen Y. (2017). CSFV infection up-regulates the unfolded protein response to promote its replication. Front. Microbiol..

[164] Versteeg G.A., Nes P.S.v.d., Bredenbeek P.J., Spaan W.J.M. (2007). The coronavirus spike protein induces endoplasmic reticulum stress and upregulation of intracellular chemokine mRNA concentrations. J. Virol..

[165] Fung T.S., Liao Y., Liu D.X. (2014). The endoplasmic reticulum stress sensor IRE1α protects cells from apoptosis induced by the coronavirus infectious bronchitis virus. J. Virol..

[166] Xue M., Fu F., Ma Y., Zhang X., Li L., Feng L. (2018). The PERK arm of the unfolded protein response negatively regulates transmissible gastroenteritis virus replication by suppressing protein translation and promoting type I interferon production. J. Virol..

[167] Chen Y.M., Gabler N.K., Burrough E.R. (2022). Porcine epidemic diarrhea virus infection induces endoplasmic reticulum stress and unfolded protein response in jejunal epithelial cells of weaned pigs. Vet Pathol..

[168] Wang Y., Li J.R., Sun M.X., Ni B., Huan C., Huang L. (2014). Triggering unfolded protein response by 2-Deoxy-D-glucose inhibits porcine epidemic diarrhea virus propagation. Antiviral Res..

[169] Favreau D.J., Desforges M., St-Jean J.R., Talbot P.J. (2009). A human coronavirus OC43 variant harboring persistence-associated mutations in the S glycoprotein differentially induces the unfolded protein response in human neurons as compared to wild-type virus. Virology.

[170] Fang P., Tian L., Zhang H., Xia S., Ding T., Zhu X. (2022). Induction and modulation of the unfolded protein response during porcine deltacoronavirus infection. Vet. Microbiol..

[171] Sims A.C., Mitchell H.D., Gralinski L.E., Kyle J.E., Burnum-Johnson K.E., Lam M. (2021). Unfolded protein response inhibition reduces middle east respiratory syndrome coronavirus-induced acute lung injury. mBio..

[172] Gao P., Chai Y., Song J., Liu T., Chen P., Zhou L. (2019). Reprogramming the unfolded protein response for replication by porcine reproductive and respiratory syndrome virus. PLoS Pathog..

[173] Rohde C., Pfeiffer S., Baumgart S., Becker S., Krähling V. (2023). Ebola virus activates IRE1α-dependent *XBP1u* splicing. Viruses.

[174] Rohde C., Becker S., Krähling V. (2019). Marburg virus regulates the IRE1/XBP1-dependent unfolded protein response to ensure efficient viral replication. Emerg. Microbes. Infect..

[175] Hassan I.H., Zhang M.S., Powers L.S., Shao J.Q., Baltrusaitis J., Rutkowski D.T. (2012). Influenza A viral replication is blocked by inhibition of the inositol-requiring enzyme 1 (IRE1) stress pathway. J. Biol. Chem..

[176] Leão T.L., Lourenço K.L., de Oliveira Queiroz C., Serufo Â.V., da Silva A.M., Barbosa-Stancioli E.F. (2023). Vaccinia virus induces endoplasmic reticulum stress and activates unfolded protein responses through the ATF6α transcription factor. Virol. J..

[177] Isler J.A., Skalet A.H., Alwine J.C. (2005). Human cytomegalovirus infection activates and regulates the unfolded protein response. J. Virol..

[178] Gilardini Montani M.S., Falcinelli L., Santarelli R., Granato M., Romeo M.A., Cecere N. (2020). KSHV infection skews macrophage polarisation towards M2-like/TAM and activates Ire1 α-XBP1 axis up-regulating pro-tumorigenic cytokine release and PD-L1 expression. Br. J. Cancer..

[179] Johnston B.P., McCormick C. (2020). Herpesviruses and the unfolded protein response. Viruses.

[180] Burnett H.F., Audas T.E., Liang G., Lu R.R. (2012). Herpes simplex virus-1 disarms the unfolded protein response in the early stages of infection. Cell Stress Chaperones..

[181] Carpenter J.E., Jackson W., Benetti L., Grose C. (2011). Autophagosome formation during varicella-zoster virus infection following endoplasmic reticulum stress and the unfolded protein response. J. Virol..

[182] Gao P., Ren J., Zhou Q., Chen P., Zhang A., Zhang Y. (2025). Pseudorabies virus inhibits the unfolded protein response for viral replication during the late stages of infection. Vet. Microbiol..

[183] Lazar C., Uta M., Branza-Nichita N. (2014). Modulation of the unfolded protein response by the human hepatitis B virus. Front. Microbiol..

[184] Hou L., Dong J., Zhu S., Yuan F., Wei L., Wang J. (2019). Seneca valley virus activates autophagy through the PERK and ATF6 UPR pathways. Virology.

[185] Cheng J.-H., Sun Y.-J., Zhang F.-Q., Zhang X.-R., Qiu X.-S., Yu L.-P. (2016). Newcastle disease virus NP and P proteins induce autophagy via the endoplasmic reticulum stress-related unfolded protein response. Sci. Rep..

[186] Tripathi A., Iyer K., Mitra D. (2023). HIV-1 replication requires optimal activation of the unfolded protein response. FEBS Lett..

[187] Taylor G.M., Raghuwanshi S.K., Rowe D.T., Wadowsky R.M., Rosendorff A. (2011). Endoplasmic reticulum stress causes EBV lytic replication. Blood.

[188] Gaguancela O.A., Zúñiga L.P., Arias A.V., Halterman D., Flores F.J., Johansen I.E. (2016). The IRE1/bZIP60 pathway and bax inhibitor 1 suppress systemic accumulation of potyviruses and potexviruses in *Arabidopsis* and *Nicotiana benthamiana* plants. Mol. Plant Microbe Interact..

[189] Gayral M, Arias Gaguancela O, Vasquez E, Herath V, Flores F.J, Dickman M.B (2020). Multiple ER-to-nucleus stress signaling pathways are activated during plantago asiatica mosaic virus and turnip mosaic virus infection in *Arabidopsis thaliana*. *Plant J.*.

[190] Guo Z., Jiang N., Li M., Guo H., Liu Q., Qin X. (2024). A vicinal oxygen chelate protein facilitates viral infection by triggering the unfolded protein response in *Nicotiana benthamiana*. J Integr Plant Biol..

[191] Lu Y., Yin M., Wang X., Chen B., Yang X., Peng J. (2016). The unfolded protein response and programmed cell death are induced by expression of garlic virus X p11 in *Nicotiana benthamiana*. J. Gen. Virol..

[192] Sun Z., Yang D., Xie L., Sun L., Zhang S., Zhu Q. (2013). Rice black-streaked dwarf virus P10 induces membranous structures at the ER and elicits the unfolded protein response in *Nicotiana benthamiana*. Virology.

[193] Li F.F., Sun H.J., Jiao Y.B., Wang F.L., Yang J.G., Shen L.L. (2018). Viral infection-induced endoplasmic reticulum stress and a membrane-associated transcription factor NbNAC089 are involved in resistance to virus in *Nicotiana benthamiana*. Plant Pathol..

[194] Qiao W., Helpio E.L., Falk B.W. (2018). Two crinivirus-conserved small proteins, P5 and P9, are indispensable for efficient lettuce infectious yellows virus infectivity in plants. Viruses.

